# Multiple functions of CREB-binding protein during postembryonic development: identification of target genes

**DOI:** 10.1186/s12864-017-4373-3

**Published:** 2017-12-29

**Authors:** Amit Roy, Smitha George, Subba Reddy Palli

**Affiliations:** 1 Department of Entomology, College of Agriculture, University of Kentucky, Lexington, KY 40546 USA; 2 Present address, Faculty of Forestry and Wood Sciences, EXTEMIT-K, Czech University of Life Sciences, Kamýcká 1176, Prague 6, 165 21 Suchdol, Czech Republic

**Keywords:** *Tribolium*, Kr-h1, CBP, RNAi, RNA seq, Juvenile hormone, Ecdysone

## Abstract

**Background:**

Juvenile hormones (JH) and ecdysteroids control postembryonic development in insects. They serve as valuable targets for pest management. Hence, understanding the molecular mechanisms of their action is of crucial importance. CREB-binding protein (CBP) is a universal transcriptional co-regulator. It controls the expression of several genes including those from hormone signaling pathways through co-activation of many transcription factors. However, the role of CBP during postembryonic development in insects is not well understood. Therefore, we have studied the role of CBP in postembryonic development in *Tribolium*, a model coleopteran insect.

**Results:**

CBP is ubiquitously expressed in the red flour beetle, *Tribolium castaneum.* RNA interference (RNAi) mediated knockdown of CBP resulted in a decrease in JH induction of Kr-h1 gene expression in *Tribolium* larvae and led to a block in their development. Moreover, the injection of CBP double-stranded RNA (dsRNA) showed lethal phenotypes within 8 days of injection. RNA-seq and subsequent differential gene expression analysis identified CBP target genes in *Tribolium*. Knockdown of CBP caused a decrease in the expression of 1306 genes coding for transcription factors and other proteins associated with growth and development. Depletion of CBP impaired the expression of several JH response genes (e.g., Kr-h1, Hairy, early trypsin) and ecdysone response genes (EcR, E74, E75, and broad complex). Further, GO enrichment analyses of the downregulated genes showed enrichment in different functions including developmental processes, pigmentation, anatomical structure development, regulation of biological and cellular processes, etc.

**Conclusion:**

These data suggest diverse but crucial roles for CBP during postembryonic development in the coleopteran model insect, *Tribolium*. It can serve as a target for RNAi mediated pest management of this stored product pest.

**Electronic supplementary material:**

The online version of this article (10.1186/s12864-017-4373-3) contains supplementary material, which is available to authorized users.

## Background

The endocrine and developmental pathways regulate insect development. Larval growth, prior to metamorphosis, consists of a number of successive instars that are punctuated by molts that are regulated by major hormones, ecdysteroids and Juvenile Hormones (JH). 20- hydroxyecdysone (20E) is the major functional ecdysteroid that regulates molting and metamorphosis [1]. 20E binds to ecdysteroid receptor (EcR) and Ultraspiracle (USP) in target tissues leading to an upregulation of ecdysteroid-inducible genes including E75, E74, E93 and BRC [2–4]. Interestingly, most of these 20E inducible genes encode transcription factors that further regulate expression of downstream genes.

Insect juvenile hormone (JH) also regulates many aspects of insect life including reproduction and development. Recently, a bHLH transcription factor methoprene-tolerant (Met) is identified as a JH receptor in the fruit fly, *Drosophila melanogaster* and other model insects including *Bombyx mori*, *Aedes aegypti, Blattella germanica* and *Tribolium castaneum* (see references [5, 6] for review). Further studies on JH signaling pathway revealed mechanisms of JH action as well as its cross-talk with 20-hydroxyecdysone (20E), insulin signaling and WNT pathways [7–15]. Hundreds of genes regulated by JH have been identified in these insects, and one gene consistently identified as an important player in JH action is krüppel homolog 1 (Kr-h1) [16–21]. The Kr-h1 expression is regulated by both JH and 20-hydroxyecdysone [22, 23]. The expression of Kr-h1 is directly induced by JH through Met, steroid receptor co-activator (SRC) and juvenile hormone response elements (JHRE) present in the promoter region [18, 24, 25].

Significant advances have been made in insect endocrinology research during the past few years. These studies have led to the discovery of major players including EcR, USP, E75, E73, E93, BRC, Met, Kr-h1 in 20E and JH signaling pathways [2–4, 6]. However, the regulatory mechanisms triggering such an orchestrated expression of different genes, transcription factors, coactivators, and repressors during the molting and metamorphosis in insects are not yet fully understood. Specifically, our knowledge regarding the molecular mechanisms underlying JH action is still limited.

CREB-binding protein (CBP) and its vertebrate paralog p300 are considered as one of the most promiscuous transcriptional co-regulator discovered to date with participation in the activities of hundreds of different transcription factors [26]. In addition to other properties, CBP and p300 have histone acetyltransferase (HAT) activity and act as hubs in transcription networking with more than 400 interaction partners, most of them are transcription factors and growth regulators [27, 28]. Thus, CBP is an important player in the development and may be involved in hormone signaling pathways in insects. In *Drosophila melanogaster*, the CBP homolog known as Nejire regulates embryonic segmental polarity during embryogenesis through Hedgehog and Wingless signaling pathways [29, 30] and dorsal-ventral patterning through the TGF-β signaling pathway [31].

During the postembryonic development in *Drosophila*, the contribution of Nejire to ecdysone signaling pathway has been reported. CBP was shown to play critical roles in initiating dendrite pruning. The HAT activity of CBP involved in acetylation of H3K27 is required for sox14 expression, which is an ecdysone response gene [32]. Acetylated H3K23 is localized to the promoters of Eip74EF and Eip75B, the 20E-induced transcription factors that play key roles in ecdysteroid action, and the acetylation levels of H3K23 correlate with the 20E-induced expression of these genes. Here also, acetylation is promoted by Nejire, the CBP homolog [33]. In *Drosophila*, lysine acetylation sites have been identified in the proteome using high-resolution mass spectrometry [34]. The sites of such modifications are highly conserved between humans and fruit flies. Furthermore, a study comparing lysine acetylation sites among fruit fly, human, nematode and zebrafish showed more conservation in the acetylated lysine residues than in the non-acetylated lysine residues [34].

However, there is limited information on the role of CBP or Nejire in postembryonic development of insects. Specifically, the function of CBP in JH action is not well studied. Recently, Fernandez et al., [35] showed that CBP plays a critical role in the regulation of metamorphosis in *Blattella*. In the current study, we employed RNAi and RNA sequencing to determine the function of CBP in the model insect, the red flour beetle, *Tribolium*.

## Results

### CBP expression in *Tribolium larvae*

**Fig. 1:**
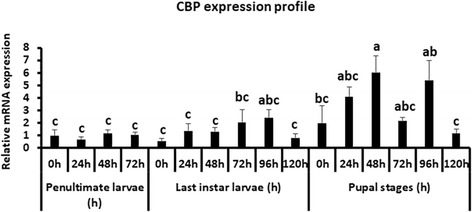
Relative CBP mRNA levels in *Tribolium* determined by qRT-PCR. Samples were collected at 24 h intervals during penultimate larvae, final instar larvae and pupal stages. Total RNA was extracted from pools of two larvae/pupae for each time period and converted to cDNA. The cDNA and gene-specific primers were used to quantify CBP mRNA levels using Ribosomal Protein 49 (Rp49) mRNA levels for normalization. The CBP mRNA levels in newly molted penultimate larvae were set as 1. Mean + SE of four replications were shown (Letters represent significance at 95% CI)

To study the expression of CBP gene during larval development, whole larvae and pupae were collected for RNA extraction at 24 h intervals during penultimate and final instar larval and pupal stages. qRT-PCR was performed with gene specific primers (Additional file 1: Table S1). The CBP mRNA was detected in all the stages tested (Fig. 1). The CBP mRNA levels were lower during the penultimate instar and early stages of the final instar, then the mRNA levels gradually increased and reached the maximum levels at 48 h after metamorphosis into the pupal stage. In general, higher levels of CBP mRNA were detected during the pupal stage when compared to its levels during the larval stages (Fig. 1).

### CBP RNAi affects growth and induces mortality

**Fig. 2:**
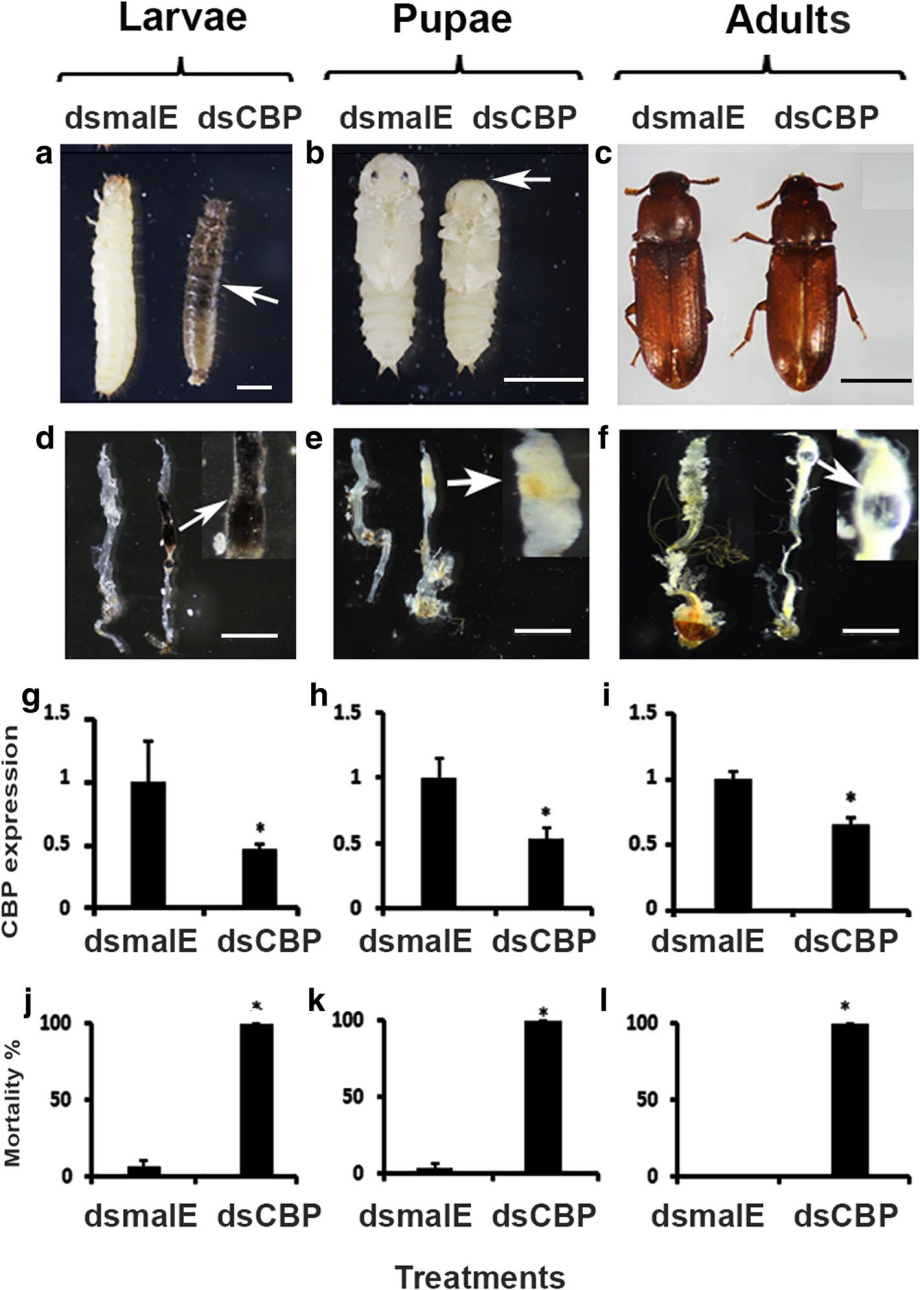
CBP knockdown causes lethality in larvae, pupae and adults of *Tribolium*. dsCBP was injected into newly molted last instar larvae, newly formed pupae and newly emerged adults. Phenotypes observed after 4 days after dsCBP injection into larvae (**a**), pupae (**b**) and adults (**c**) respectively. Dissected alimentary canals (mostly midgut region) from dsmalE and dsCBP injected larvae (**d**), pupae (**e**) and adults (**f**). Arrows point to the enlarged areas of the alimentary canals that showed darkened regions. Knockdown efficiency at 24 h after dsCBP injection in larvae (**g**), pupae (**h**) and adults (**i**). Percent mortality observed at 8 days after dsCBP injection into larvae (**j**), pupae (**k**) and adults (**l**). Scale Bar: 1 mm. [One way AVOVA, * *P* < 0.05]

To understand the role of CBP in the regulation of larval and pupal development and metamorphosis, we employed RNAi mediated gene knockdown. The CBP double-stranded RNA (dsCBP) was injected into the newly molted last instar larvae, newly formed pupae, and newly emerged adults. Mortality and developmental defects were recorded. By about 4 days after the injection of dsCBP, the final instar larvae stopped feeding and growing, and remained smaller and turned dark brown color when compared to the control larvae injected with dsmalE (Fig. 2a). The CBP knockdown caused an arrest in development of the larvae during the quiescent stage prior to metamorphosis into pupal stage. The dsCBP injected larvae remained small and died after a few days. The dsCBP injected larvae also showed dark melanized areas in the midgut region of the alimentary canal (Fig. 2d). The dsmalE injected control larvae grew normally with no melanized areas in the midgut region of the alimentary canal and pupated after 5 days. Injection of dsCBP resulted in a significant decrease in CBP mRNA levels and caused 100% mortality (Fig. 2g&j). Injection of dsCBP into newly formed pupae blocked growth and development of pupae as well. The differentiation of compound eyes was impaired by depletion of CBP expression in newly formed pupae. Furthermore, pupal size was reduced in dsCBP-injected pupae, and the midgut region of the alimentary canal developed dark yellowish areas (Fig. 2b&e). The dsmalE-injected pupae emerged as normal adults in about 5 days after treatment. Injection of dsCBP into newly formed pupae resulted in a significant decrease in CBP mRNA levels and caused 100% mortality (Fig. 2h&k). Injection of dsCBP into newly emerged adults did not show any distinct phenotype after 4 days (Fig. 2c). Interestingly, the alimentary canal dissected from the CBP RNAi adults showed dark areas in the midgut region similar those observed in the larvae and pupae (Fig. 2f). Injection of dsCBP into newly formed adults resulted in a significant decrease in CBP mRNA levels and caused 100% mortality (Fig. 2i&l).

### CBP is required for expression of JH-response gene, Kr-h1

**Fig. 3:**
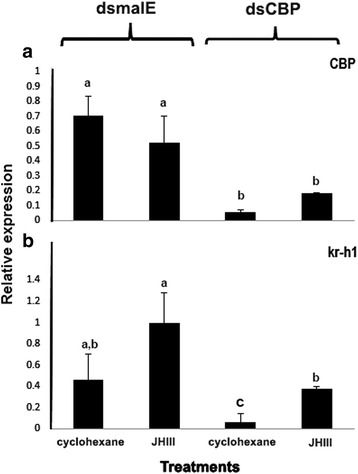
CBP is required for expression of Kr-h1 in *Tribolium* larvae. The newly emerged final instar larvae were injected with 1 μg of either dsmalE or dsCBP. Seventy-two hours later, 0.5 μl of 10 μM hydroprene or cyclohexane (control) was topically applied. Six hours after the application, total RNA was isolated and used to quantify mRNA levels of CBP (**a**) and Kr-h1 (**b**) in each treatment by qRT-PCR using RP49 as a control. The data shown are M + SE (*n* = 3). (Letters represent significance at 95% CI)

To determine whether CBP is required for JH-induced gene expression in the larvae, CBP was knockdown by injection of dsCBP into the newly ecdysed final instar larvae. Because the endogenous JH III levels are low in day 3 final instar larvae [36], the JH analog, hydroprene, was topically applied to these insects at 72 h after injection of dsCBP or control dsmalE. For control, cyclohexane was applied on dsmalE and dsCBP injected larvae. At 6 h after application of hydroprene or cyclohexane, the total RNA was isolated and utilized to determine relative mRNA levels using qRT-PCR. The CBP mRNA levels decreased by about 70% in dsCBP injected insects when compared to its levels in control insects injected with dsmalE (Fig. 3a). A significant reduction in Kr-h1 mRNA levels was detected in larvae injected with dsCBP when compared to its levels in control larvae injected with dsmalE after both cyclohexane and hydroprene treatments (Fig. 3b). These results showed that CBP is required for Kr-h1 expression in the larvae.

### Differential gene expression (DGE) analysis in CBP knockdown larvae

**Fig. 4:**
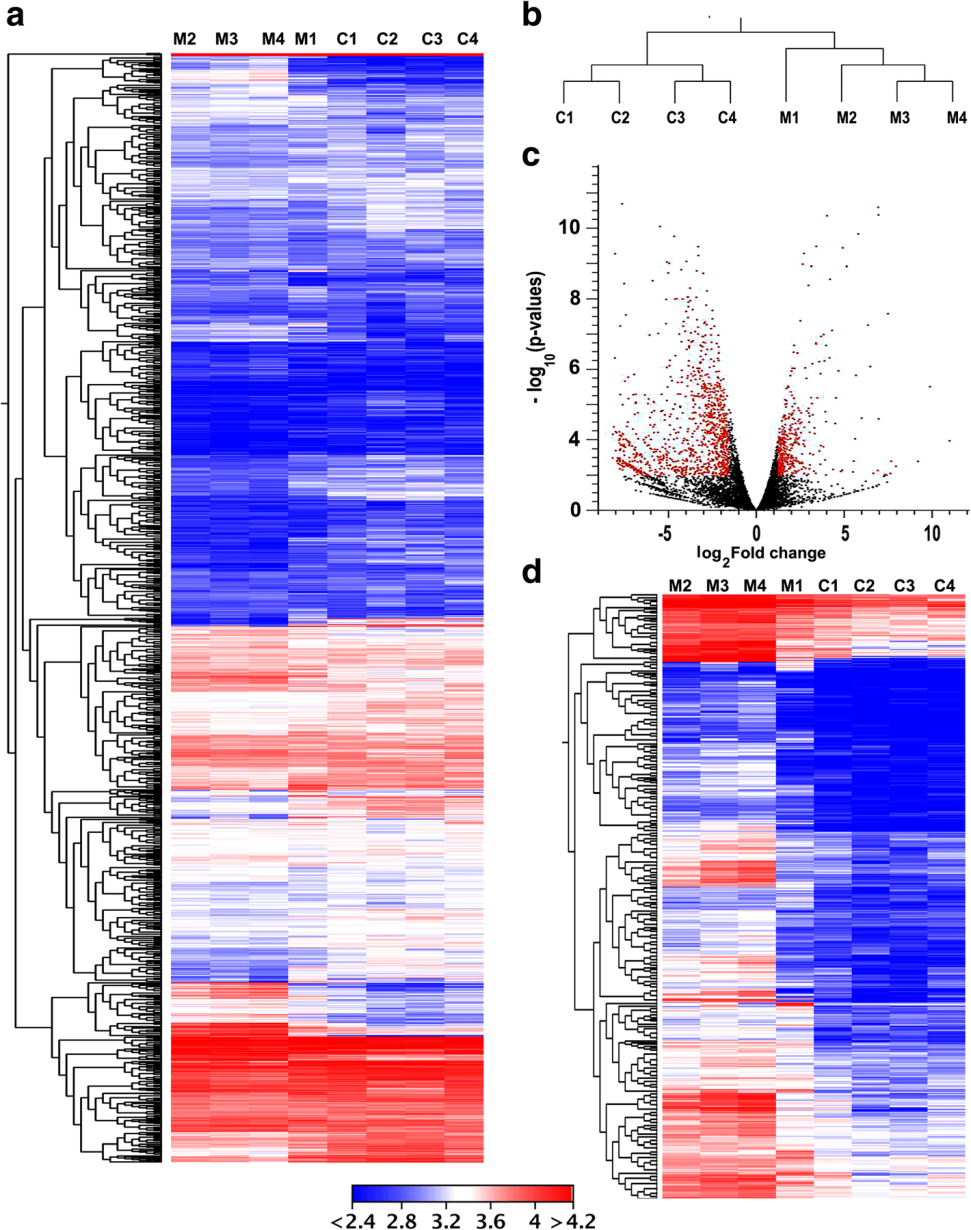
Gene expression analysis of RNA isolated from dsmalE (control, M1-M4) and dsCBP injected (C1-C4) last instar *Tribolium* larvae using the empirical analysis of DGE (EDGE) algorithm in CLC Genomics Workbench (Version: 9.5.9) and WEGO. **a** Heatmap showing the overall gene expression pattern of all four biological replicates from malE or CBP dsRNA injected larvae. The color spectrum, stretching from blue to red, represents TMM normalized expression values obtained after EDGE analysis. **b** Hierarchical clustering based on the overall gene expression pattern showing the relative differences within and among the biological replicates from malE or CBP dsRNA injected larvae. **c** Volcano plot of expression data after EDGE analysis. In the plot, log_2_ of fold change between the malE and CBP dsRNA-treated insects are plotted against −log10 (p), where p is a probability value (for a given gene) that is associated with the EDGE comparison of the two groups of samples. The red dots indicate the number of significantly up- and down-regulated genes using *p* < 0.01 and ±2-fold change as the cutoff threshold. **d** Heatmap illustrating the TMM normalized expression values of 1306 significantly downregulated genes (*p* < 0.01, ≤2-fold ↓) after CBP knockdown in *Tribolium* larvae. Color-coding is the same as in figure (**a**)

**Fig. 5:**
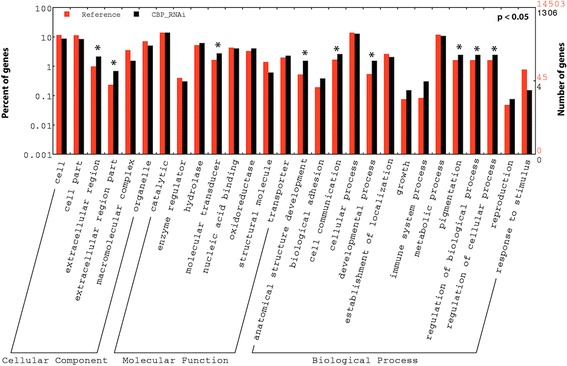
GO enrichment analysis. WEGO output for GO enrichment analysis with 1306 genes that were downregulated in *Tribolium* larvae after CBP knockdown. The plot shows percent and number of overrepresented GO terms compared to the reference genome (*Tribolium* genome). Asterisks indicated the statistically significant (*p* < 0.05) enrichment of important GO categories mainly under cellular component, molecular function and biological process categories after CBP RNAi

**Fig. 6:**
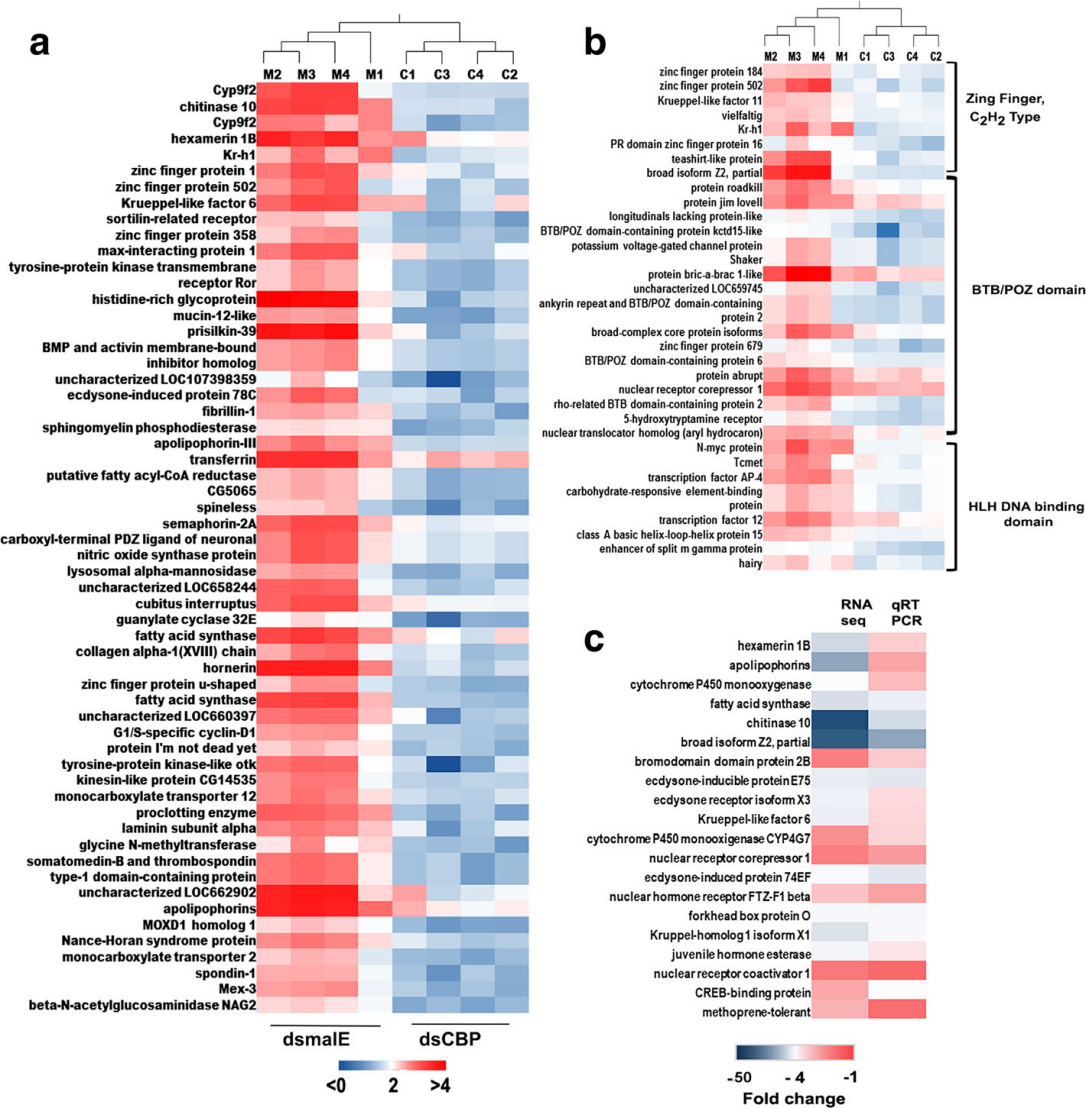
Post-EDGE data analysis. **a** Heatmap representing the expression levels of a subset of 53 genes in malE and CBP dsRNA-treated samples that clustered together with Kr-h1 after K-mer featured clustering of the data set containing 1306 downregulated genes in larvae injected with CBP dsRNA. **b** Heatmap showing the expression profile of Zinc finger, BTB/POZ, and HLH DNA-binding domain-containing genes in *Tribolium* larvae injected with CBP dsRNA. **c** Validation of RNA seq expression data by qRT-PCR using a subset of 20 genes. The fold change values obtained by qRT-PCR and RNA seq for each gene are plotted as a heatmap. Four biological replicates for each treatment (malE and CBP) were used for qRT-PCR analysis, and the mean fold change value of each gene was used in the heatmap. The experiment was repeated twice with comparable results

To identify genes regulated by CBP, newly molted *Tribolium* last instar larvae were injected with dsmalE (control) or dsCBP, RNA isolated was from the larvae at 12 h after dsRNA injection and used for library preparation and sequencing. Illumina HiSeq4000 sequencing resulted in 147.9 million reads (i.e., 28.2, 31.7, 50.7, 37.2 million) for control samples and 109.6 million reads (i.e.,25.7, 22.3, 34.3, 27.2 million) for CBP knockdown samples with over 21.3 billion bp sequence information altogether and an estimated transcriptome coverage ranging from 23× to 52× for each of the four biological replicates. After quality control measures, 110.6 million reads (~94%) from control samples and 75.8 million reads (~88%) from CBP knockdown samples were mapped back to the reference genome of *Tribolium* (Table 1). After RPKM-based normalization of the gene expression, DGE was performed using the EDGE analysis tool, and the overall pattern of gene expression in control and CBP knockdown samples was represented as a heatmap (Fig. 4a). The expression differences within the replicates of treatment are smaller than between the treatments, thus, the replicates of each treatment cluster together (Fig. 4b). Differentially expressed genes are shown as a volcano plot with red dots indicating the statistically significant (*p* < 0.01 and ≥2-fold) expression differences between CBP (treatment) and malE (control) dsRNA-injected larvae (Fig. 4c). The expression levels of CBP in the larvae injected with dsCBP were >1.5-fold (EDGE test, *p* < 0.02) lower when compared to that of the control larvae. Approximately 1800 genes were differentially expressed, and of them, 1306 were significantly downregulated (*p* < 0.01, ≤2-fold) after CBP RNAi (Fig. 4d). Details on the Blast2GO hits in the NR databases, hit accessions, functional annotations, and the relative expression levels of these 1306 downregulated genes across the respective RNA seq samples can be found in Additional file 1: Figure S3 and Additional file 2: Excel file S1. Many of these downregulated genes have epifactor domains (Additional file 1: Figure S4) and belong to signaling pathways including FoxO, WNT, and mTOR (Additional file 1: Figure S5 and Supporting information S1). Also, GO enrichment analyses of 1306 downregulated genes revealed considerable enrichment in different molecular functions including oxidoreductase activity, hydrolase activity, transporter activity and molecular transducer activity. Biological processes such as developmental process, immune response, pigmentation, reproduction and regulation of cellular process were also enriched among the downregulated genes (Fig. 5). We identified 52 genes by K-mer cluster analysis that showed similar expression profile as Kr-h1, one of the important players in JH action (Fig. 6a, Additional file 3: Excel file S2). CBP RNAi also affected the expression of several genes with DNA-binding domains such as C2H2-type Zinc finger, BTB/PAZ, and bHLH that are known to play significant roles in insect development (Fig. 6b).

**Table 1:** RNA-seq statistics

A. Run Summary:-								
Lane	PF* Yield (bp)		Number of PF* Clusters**		Q30%		Average Quality Score	
8	21,318,423,944		318,185,432		84.88		35.60	
*Passed Filter (PF). **For single read sequencing (SR), number of reads = number of clusters								
B.								
	dsmalE				dsCBP			
Sample	M1	M2	M3	M4	C1	C2	C3	C4
Total no. of reads	28,222,259	31,75,2000	50,71,6160	37,289,035	25,719,392	22,313,906	34,360,910	27,296,096
No. of high-quality reads	23,363,204	25,935,799	36,008,785	25,162,378	20,027,446	17,999,025	19,243,770	18,616,332
Percent reads mapped back to *Tribolium* genome	91.51	95.94	94.97	94.41	91.01	90.05	83.28	87.73

(A) Table showing the sequencing run summary of the pooled cDNA libraries prepared using RNA isolated from larvae injected with malE and CBP dsRNA. Total 318,185,432 reads obtained from all samples with an average quality score of 35.60. (B) Summary of read statistics after Illumina Hi-seq 4000 sequencing. Total reads contain the raw number of reads obtained after de-multiplexing of total reads (318,185,432) from the sample lane (8 sample/lane) from Illumina Hi-seq 4000 sequencing. Number of high quality reads indicates the number of reads left after read trimming and filtering (Quality control step). Percentage of high quality reads unambiguously mapped to reference *Tribolium* genome are shown in the bottom row

### qRT-PCR validation

**Fig. 7:**
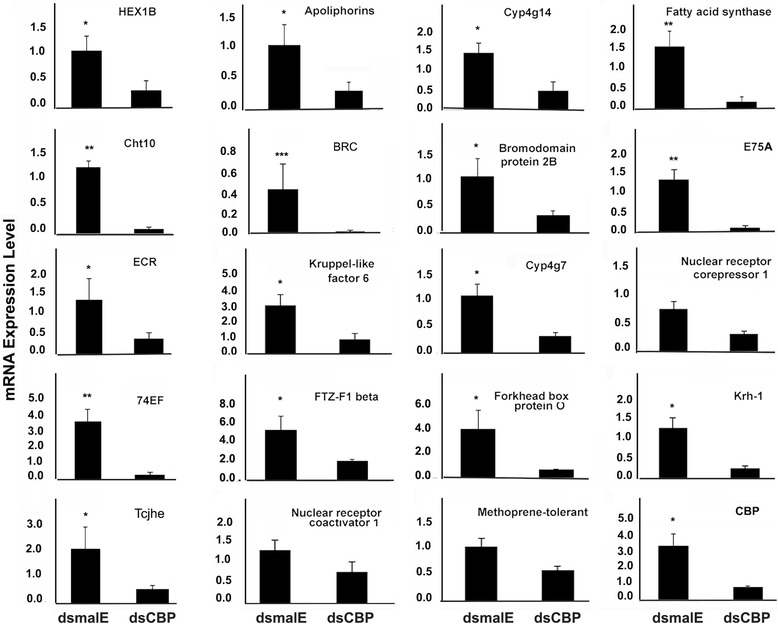
Relative mRNA levels of a subset of 20 genes selected from the downregulated genes in dsCBP-injected larvae. Relative mRNA levels of these genes were determined by qRT-PCR as described in Fig. 1 legend. Each panel represents the relative mRNA levels of one target gene in dsmalE and dsCBP treated samples. Mean ± S.E (*n* = 4) are shown. [One-Way AVOVA, * *P* < 0.05, ** *P* < 0.01, *** *P* < 0.001]

The data on the relative expression of the selected subset of 20 genes using qRT-PCR showed a positive correlation with the RNA seq data (Fig. 6c and Additional file 1: Figure S6). For example, a 5 and 13-fold decrease in Kr-h1 mRNA levels were detected in CBP knockdown larvae by qRT-PCR and RNA seq analyses respectively. The trends of gene expression are the same between the RNA seq and qRT-PCR analyses, but the magnitude of the change is different due to the varied sensitivity of the analysis platforms. The qRT-PCR did not detect any statistically significant change in the expression of TcMet, but RNA seq data predicted a 2.8-fold decrease (*p* < 0.01) in TcMet expression in CBP knockdown larvae. The details on the comparison of the expression levels of 20 selected genes by qRT-PCR and RNA seq are shown in Fig. 7.

## Discussion

More than two decades have passed since the discovery of CBP as a transcriptional co-activator due to the presence multiple protein binding domains [37]. However, in insects, most of the studies on this transcriptional coregulator, CBP, are restricted to the model insect, *Drosophila* and its embryonic stage [29–31, 38–41]. There is limited information available about its impact on postembryonic development in insects. Recently, Fernandez-Nicolas et al., have reported on the contribution of CBP to the postembryonic development of *Blattella*. Our studies have extended the effort in the similar direction by examining the involvement of CBP in postembryonic development of a coleopteran model insect, *Tribolium.* CBP is ubiquitously expressed in the red flour beetle. The stage specific expression levels suggest that CBP is necessary during all stages, especially during quiescent and pupal stages (Fig. 1). Knockdown of CBP by RNAi is lethal during all beetle life stages tested, which indeed account for the fundamental role of CBP in *Tribolium*. Furthermore, interesting phenotypes have been observed in CBP RNAi larvae i.e., reduced growth and increase in melanization in the alimentary canal. The JH driven increase in the expression of Kr-h1, a major contributor to JH-dependent antimetamorphic action [19], has also decreased after injection of dsCBP indicating the plausible function of CBP as a coactivator in JH response gene expression (Fig. 3b).

Gene expression is a multi-layered process, regulated by many factors including histone and DNA modifiers, DNA-binding proteins, small RNAs, post-translational modifiers of nuclear proteins, and proteins involved in nuclear-cytoplasmic shuttling of transcription factors [24, 42–45]. Gene activation involves stripping of some or all of the silencing components from the promoter regions and recruiting proteins including activators and co-activators involved in transcription. Knockdown of CBP has caused significant downregulation of 1306 genes in *Tribolium* larvae. Many of these genes are associated with cellular and biological process regulation, pigmentation, developmental processes including anatomical structure development, and other key regulatory functions as revealed by GO enrichment analysis. Moreover, CBP knockdown also affected the cellular immune response, pigmentation, and metabolic process, which may have caused the enhanced melanization areas detected in the midgut of CBP knockdown animals (Fig. 2).

### Depletion of CBP impairs hormone signaling

#### JH signaling

The presence of JH and expression of JH-response gene, Kr-h1, during larval development ensures molting whereas a drop in JH levels and expression of Kr-h1 induce metamorphosis in the presence of ecdysteroids. In *Tribolium*, Kr-h1 levels decrease at the end of the larval stage due to a decrease in JH biosynthesis, which leads to initiation of metamorphosis [19]. In this insect, application of JH analog, hydroprene extends larval stage and induce supernumerary larval molts [46] The Kr-h1 mRNA levels decreased by 13-fold in CBP knockdown larvae compared to that in the control larvae, further confirming a role for CBP in larval growth and metamorphosis. In *D.melanogaster*, the Kr-h1 mediates JH action during larval stages and 20E signaling during larval-pupal metamorphosis [23, 47–49]. Kr-h1 is a transcription factor containing a DNA-binding domain consisting of C_2_H_2_ zinc finger motifs and amino acid spacers connecting them [50]. Genes coding for seven additional C_2_H_2_ zinc finger domain-containing proteins including BRC are also downregulated in CBP knockdown larvae (Fig. 6b). The BRC proteins are coded by one of the early ecdysone response genes [51] that codes for a complex of proteins involved gene regulation during insect development and works as a key specifier of the pupal commitment [52, 53]. Interestingly, JH can also regulate BRC expression and is inducible by exogenous application of JH mimic during pupal stage [54–56]. Normally, BRC expression in *Tribolium* increases at the end of final instar larval stage following a reduction in Kr-h1 expression [54–57]. Another JH target gene, hairy, is also affected by CBP RNAi and the role of hairy as a target gene of Met in the JH gene repression hierarchy in *Aedes aegypti* has been reported recently by Saha et al., [58]. Moreover, early trypsin genes whose expression is also controlled by JH [59] are downregulated in CBP RNAi larvae. The expression of trypsin-1, trypsin-7 and trypsin II-P29 are reduced by 174, 128 and 29-fold respectively in CBP knockdown larvae when compared to their expression in control larvae (Additional file 2: Excel file S1). However, the expression of TcSRC is unaffected by CBP RNAi. TcSRC and TcMet co-regulate expression of several JH response genes in *Tribolium* [10]. The RNA seq analysis predicted downregulation of Met in CBP RNAi larvae. However, a decrease in TcMet expression in CBP RNAi larvae was not observed in the qRT-PCR experiment. TcMet is a JH receptor and contains a basic helix-loop-helix DNA-binding domain (bHLH DNA-binding domain). Genes coding for other bHLH DNA-binding domain-containing proteins including N-myc, AP-4, and tango, the aryl hydrocarbon receptor nuclear translocator homolog identified previously in *Tribolium* [60] are downregulated in CBP RNAi larvae. N-myc controls cell fate by regulating gene transcription, and the CBP was shown to act as a Myc co-activator and stimulates Myc-dependent transcription of gene expression in a HAT domain-dependent manner [61].

#### Ecdysone signaling

Depletion of CBP affected ecdysone-inducible genes such as ecdysone receptor (EcR), E74, E75, and BRC. EcR is one of the key players in ecdysone signaling cascade. 20E binds to its receptor EcR and induces the expression a series of early and early-late response genes that codes for additional transcription factors that control gene expression cascades [62]. The activity of EcR in dendrite pruning in *Drosophila* nerves is affected by CBP [32]. One possible explanation for these results is that the CBP indeed affects the activity of EcR similar to that in steroid receptors described in mammals [63]. The CBP may also interfere with the ecdysteroid biosynthesis as reported for steroid biosynthesis in vertebrates by modulating the expression of Cytochrome P450 enzymes involved in steroid hormone biosynthesis [64, 65]. The expression of genes coding for multiple Cytochrome p450 family members is also affected by CBP knockdown. One of them is CYP307B1, a member of the Halloween family that is involved in ecdysteroid biosynthesis [66]. A few other members of the CYP4 family (CYP4Q1, CYP4Q2, and CYP4Q7) are suppressed by CBP RNAi in *Tribolium*. The CYP4 group enzymes are known to be involved in the synthesis and metabolism of endogenous hormones and other compounds [67].

The E74 is induced directly by ecdysone and encodes transcription factors regulating ecdysone primary- and secondary-response genes [68]. *E75* plays an important role in JH and ecdysone crosstalk and codes for four isoforms [69]. E75A is one of the isoforms with the complete DNA-binding domain, and CBP RNAi suppressed its expression. E75A, one of the transcriptional targets of JH, regulates the timing of metamorphosis in *Drosophila* by suppressing the expression of BRC during the early instars [70]. It is also expressed during each of the ecdysone pulses that trigger larval molting [71]. Moreover, E75A protein functions as a transcriptional repressor in vivo targeting ecdysone inducible gene, FTZ-F1 (Fushi tarazu binding factor 1), a nuclear hormone receptor, and a possible component of JH signaling pathway [71, 72]. Similar findings are documented by Fernandez-Nicolas et al., where CBP depletion affects the expression of E75A, E75B and HR3A genes from early ecdysone signaling cascade in *Blattella*. The observed delayed molting in CBP RNAi *B. germanica* larvae was predicted due to the reduced expression of above-mentioned genes. In the present study, the expression of HR3, HR4 and HR39 were reduced by 11, 8 and 3-fold after dsCBP injection (Additional file 2: Excel file S1).

Interestingly, the gene coding for Forkhead box O (FoxO) transcription factor was also downregulated in CBP dsRNA-treated larvae. In *Bombyx mori*, 20E induces FoxO expression and its nuclear localization that in turn upregulates expression of genes coding for lipases including brummer and acid lipase-1 for promoting lipolysis in the fat body cells during molting and pupation [73]. Recently, Lin et al., [74] showed that FoxO mediates the timing of pupation by regulating the ecdysteroid biosynthesis in the red flour beetle. Hence, it can be conceivable that CBP may regulate ecdysteroid biosynthesis by controlling the expression of FoxO. In *Tribolium*, vitellogenin (Vg) gene expression is under the control of JH and FoxO [8]. Vg is one of the downstream targets of bone morphogenesis protein (BMP) branch of a TGF-β signaling pathway that is involved in regulating the timing of metamorphic molt by influencing the ecdysteroid biosynthesis in *Drosophila* [75–77]. Depletion of CBP also reduces Vg gene expression as observed in cockroach. Moreover, the GO enrichment analysis also suggested an effect of CBP knockdown on reproduction (Fig. 5).

### Impact of CBP RNAi on expression of genes involved in development

Insect growth and metamorphosis are regulated by the nutrient supply to the insect. For instance, tachykinin like peptide contributes to maintaining adequate food consumption during vitellogenesis, a period of high energy demand [78]. In the current study, tachykinin like peptide receptor 86C was downregulated (ca. 40-fold) in CBP RNAi larvae. A similar experiment in cockroaches showed less food intake and slow growth due to a reduction in tachykinin and sulfakinin expression in CBP RNAi insects. In the present study, we did not measure the effect of CBP RNAi on food intake in *Tribolium* larvae, but the reduced size of the CBP RNAi larvae suggests reduced growth.

Furthermore, using K-mer clustering, we have identified 52 out of 1306 down-regulated genes with similar expression profile as Kr-h1. Some of these 52 genes are known to play a central role in insect growth. These genes include Chitinase 10 (TcCHT10), hexamerin, apolipophorins, and fatty acid synthase. TcCHT10 contains multiple catalytic domains and plays a key role in larval molting, pupation, and adult metamorphosis [79]. Involvement of hexamerins in JH-dependent gene expression has been reported in a social insect, termite [80]. Apolipophorins serve as lipid transporters; however, Apolipophorin III, downregulated by CBP knockdown, is also known to play an important role in insect immunity [81, 82]. It is also involved in regulation of phenoloxidase system. Thus, suppression of Apolipophorin expression may upset the regulation of phenoloxidase system in *Tribolium* larvae resulting in a higher degree of melanization after the CBP knockdown (Fig. 2a, d). The present study also revealed that fatty acid synthase (FAS) expression was also regulated by TcCBP. The FAS is another important player during diapause preparation in insects and lipid synthesis [83]. The transcription of FAS is suppressed by JH and the JH receptor Met. The CBP RNAi also suppressed the expression of matrix metalloproteinase 2 (Mmp2), a protein involved in pre-pupal remodeling of the larval fat body [84].

Interestingly, genes coding for Cathepsin L, juvenile hormone esterase (JHE), solute carrier family member protein, juvenile acid O methyl transferase (JHAMT), cytochrome p450 (TC002552) that are induced by JH through Met [10], are also downregulated after CBP knockdown signifying the role of TcCBP in the expression of genes that require Met for their JH-mediated regulation.

### Does CBP function as an epigenetic modulator in endocrine signaling?

In *Drosophila*, CBP functions as a histone acetyltransferase (HAT) in the 20E regulation of dendrite pruning through acetylation of H3K27; CBP also physically interacts with EcR, USP, and Brahma [32]. Histone acetyltransferase activity is also involved in the 20E–induced expression of Eip74EF and Eip75B, and CBP-mediated acetylation of histone H3K23 is required for this action [33]. Our present study does not monitor the impact of CBP-mediated acetylation of histones on the expression of genes related to *Tribolium* growth and metamorphosis. However, genes with epi-factor domains such as SET, PHD, Jmjc, LRR, C5HC2 zing finger, and Arid are downregulated after CBP knockdown (Additional file 1: Figure S4). These domains are known to be present in epigenetic enzymes such as histone lysine methylases (HMTs), histone demethylases (HDMs), lysine metyltransferases (KMTs), and lysine demethylases (KDMs) and play important roles in hormone signaling [85]. Genes containing bromodomain (BRD) and chromodomain (CHD) that recognize acetylated histones and methylated histones respectively are also affected by CBP RNAi. Hence, it is rather clear from our study that CBP may act as an epigenetic modulator by regulating expression of many epi-factor domain-containing proteins. Such epi-factor domain-containing proteins may regulate (i.e., epigenetic regulation) the expression of many genes associated with postembryonic development in *Tribolium*. However, detailed insights into the epigenetic regulation of hormone signaling by CBP requires studies using ChiP seq based or acetylome approaches in CBP RNAi larvae. These studies are currently underway in our laboratory.

## Conclusions

CBP is expressed in all life stages of the red flour beetle. CBP RNAi causes lethal phenotypes in the larvae, which are indeed a cumulative consequence of reduced expression of many vital genes in CBP RNAi larvae. Moreover, CBP regulates the expression of a cascade of early and late response genes in JH and 20E signaling pathways by regulating expression of genes involved in the action of these hormones. Hence, CBP plays a diverse yet decisive role in the regulation of the postembryonic development in the coleopteran model insect, *Tribolium*.

## Methods

### Insect rearing

The wild-type GA-1 strain of *Tribolium* [86] were maintained on organic wheat flour with 10% yeast at 30 °C and 65 ± 5% RH inside insect rearing chambers as described by earlier co-workers [87]. The larvae and pupae were staged based on the size, color, and number of days after hatching. The final instar larvae were identified based on the untanned white cuticle, observed immediately after molting. White colored pupae were considered as newly molted pupae.

### DsRNA injection and topical application of JH analog hydroprene

The newly emerged adults (within 6 h after emergence) or final instar larvae were anesthetized with ether vapor for 4–5 min and lined on a glass slide covered with 2-sided tape. The dsRNA was injected into the dorsal side of the first or second abdominal segment using an injection needle pulled from a glass capillary tube using a needle puller (Idaho Technology, Salt Lake City, UT). About 0.8–1 μg (0.1 μl) dsRNA was injected into each new male adult, pupae or each larva. The *malE* dsRNA prepared using a fragment of *E. coli malE* gene amplified using T7 primer (TAATACGACTCACT ATAGGG) and Litmus28iMal plasmid (New England Bio labs, Ipswich, MA) as a template was used as a control. The injected beetles were removed from the slide and reared on whole-wheat flour and 10% Baker’s yeast at 30 ± 1 °C for 72 h. For hormone treatment, hydroprene (1 μl of 10 μg/μl) was topically applied to adults and larvae after 72 h exposure to dsRNA. The animals were collected at 6 h after hydroprene application.

Total RNA was isolated using the TRI reagent (Molecular Research Center Inc., Cincinnati, OH). The DNA was eliminated from the total RNA using DNase I (Ambion Inc., Austin, TX) and 1 μg of total RNA for each sample was used for cDNA synthesis. cDNAs were used as templates to amplify fragments of target genes. The PCR products were used for dsRNA synthesis. The primers used in these experiments are listed in Additional file 1: Table S1. The MEGA script RNAi kit (Ambion Inc., Austin, TX) was used for dsRNA synthesis as described previously [10].

### CBP knockdown, RNA seq library preparation, and sequencing

Final instar larvae were injected with 1 μg of malE or CBP dsRNA and maintained on organic wheat flour containing 10% yeast for 12, 24, 48 h. Larvae (four biological replicates) from each time point were collected and the total RNA was extracted from the whole larvae using the TRI reagent (Molecular Research Center Inc., Cincinnati, OH) and cDNA was synthesized using M-MLV reverse transcriptase (Thermo Fisher Scientific Inc., Waltham, MA). qRT-PCR was performed to evaluate the knockdown efficiency and its impact on JH signaling pathway using Kr-h1 gene expression as a molecular marker. Samples from CBP knockdown larvae that showed a decrease in Kr-h1 expression (12 h after injection of dsRNA, Additional file 1: Figure S1 A) were selected for RNA seq library preparation and sequencing. RNA seq libraries were prepared using 2 μg RNA per replicate per library following “Method B2” as described by Ma et al. [88]. The libraries were size selected (Additional file 1: Figure S1 B), multiplexed and sequenced on an Illumina Hi-seq 4000 sequencer (Sequencing and Genomics Technologies Center of Duke University, NC, USA). Raw sequencing data statistics are shown in Table 1.

### Mapping, annotation, and DGE analysis

Raw reads after quality control (demultiplexing, trimming, adaptor removal) were mapped back to the published reference genome of GA-2 *Tribolium* strain using pre-optimized parameters (i.e. unique exon mapping, mismatch cost = 2, insertion cost = 3, deletion cost = 3, length fraction = 0.8, similarity fraction = 0.8) in CLC Genomic Workbench (Version 9.5.9, Qiagen Bioinformatics, Valencia, CA). After counting of the mapped reads, gene expression level was normalized for different dsRNA treatments (Additional file 1: Figure S2). Only uniquely mapped exon reads were considered for downstream analysis. Differential gene expression analysis was performed using the inbuilt CLC Genomic Workbench software tool, “Empirical analysis of DGE” (EDGE) employing standard parameters. A stringent *p*-value cutoff <0.01 was employed along with a ≥2-fold change to identify the differentially expressed genes between the malE and CBP dsRNA-treated samples. Using inbuilt K-mer clustering tool of CLC genomic workbench, differentially expressed genes were grouped into few clusters based on their expression features. Genes of interest were functionally annotated using the Blast2Go Pro plugin available with the CLC Genomic Workbench and represented using Web Gene Ontology Annotation Plot (WEGO) [89]. GO enrichment analysis (level 2) was performed by plotting the GO information of the target samples against all the GOs from *Tribolium* genome in WEGO. Domain search was carried out by searching the translated protein sequences of target genes against the Pfam domain database downloaded in CLC workbench. Furthermore, pathway analysis was conducted using the KEGG pathway database resources [90]. To validate the results of RNA seq data, expression of 20 selected genes was quantified using qRT-PCR. The selection of the genes was based on their potential function (i.e., DNA binding, transcription factor activity) and plausible impact on the JH signaling pathway and insect development. During qRT-PCR, a melt curve was generated to ensure single product amplification after each run. The expression levels of the target genes were calculated using 2 ^–ΔCt^ method, with RP49 (a ribosomal protein) serving as a reference housekeeping gene. Mean 2 ^–ΔCt^ values for each gene from malE and CBP knockdown group were used for fold change calculations.

## Additional files

Additional file 1: Table S1. Sequences of primers used in the experiments. **Figure S1.** Checking the knockdown efficiency in *T.castaneum* larvae and cDNA library preparation for RNA seq. **Figure S2.** Normalization of RNA-seq data. **Figure S3.** Histogram presentation of GO ontology classification with 1306 genes that were downregulated in *T.castaneum* larvae after CBP knockdown. **Figure S4.** Epi-factor domains within the downregulated genes (1306) after CBP knockdown in *T.castaneum* larvae. **Figure S5.** KEGG pathway analysis. **Figure S6.** Correlation of gene expression levels of 20 selected genes by comparing both qPCR and RNA-seq data. Supporting Information S1. KEGG pathway analysis output. (DOCX 2490 kb)

Additional file 2: Excel file S1. Details of 1306 genes downregulated by CBP RNAi. (XLSX 495 kb)

Additional file 3: Excel file S2. Details of 52 genes identified after k-mer clustering. (XLSX 18 kb)

## Abbreviations

20E: 20- hydroxyecdysone
BMP: Bone morphogenesis protein
BRD: Bromodomain
CBP: CREB-binding protein
CHD: Chromodomain
DGE: Differential gene expression
dsRNA: double-stranded RNA
EcR: Ecdysteroid receptor
FoxO: Forkhead box O transcription factor
FTZ-F1: Fushi tarazu binding factor 1
HAT: Histone acetyltransferase
HDM: Histone demethylases
JH: Juvenile hormone
JHRE: Juvenile hormone response element
KDM: Lysine demethylases
KMT: Lysine metyltransferases
Kr-h1: Krüppel homolog 1
Met: Methoprene tolerant
qRT-PCR: quantitative reverse-transcriptase PCR
SRC: Steroid receptor co-activator
USP: Ultraspiracle
Vg: Vitellogenin
