# Correction to: Multiple functions of CREB-binding protein during postembryonic development: identification of target genes

**DOI:** 10.1186/s12864-018-4939-8

**Published:** 2018-08-06

**Authors:** Amit Roy, Smitha George, Subba Reddy Palli

**Affiliations:** 1 Department of Entomology, College of Agriculture, University of Kentucky, Lexington, KY 40546 USA; 2 Present address, Faculty of Forestry and Wood Sciences, EXTEMIT-K, Czech University of Life Sciences, Kamýcká 1176, Prague 6, 165 21 Suchdol, Czech Republic

## Correction

Following the publication of this article [1], the authors found that some of the primers listed in Table 1 are not correct. This mistake occurred during assembly of the primer table and the authors apologize for this error. This correction does not change the data included in the paper, their interpretation nor the conclusions drawn.

The corrected version of “Table S1: Sequences of primers used in the experiments” is included in this Correction article (Additional file 1).

## Additional file

Additional file 1: Sequence of primers used in the experiments. **Figure S1.** Checking the knockdown efficiency in T. castaneum larvae and cDNA library preparation for RNA seq. **Figure S2.** Normalization of RNA-seq data. **Figure S3.** Histogram presentation of GO ontology classification with 1306 genes that were downregulated in T. castaneum larvae after CBP knockdown. **Figure S4.** Epi-factor domains within the downregulated genes (1306) after CBP knockdown in T. castaneum larvae. **Figure S5.** KEGG pathway analysis. Figure S6. Correlation of gene expression levels of 20 selected genes by comparing both qPCR and RNA-seq data. **Supporting Information S1.** KEGG pathway analysis output. (PDF 892 kb)
