# Supplementary material for: Multiple functions of CREB-binding protein during postembryonic development: identification of target genes
Source: BMC Genomics. 2017 Dec 29;18:996. doi: 10.1186/s12864-017-4373-3 (PMC5747157; doi:10.1186/s12864-017-4373-3)
Supplement: Supplementary file 1 — Sequences of primers used in the experiments. Figure S1. Checking the knockdown efficiency in T.castaneum larvae and cDNA library preparation for RNA seq. Figure S2. Normalization of RNA-seq data. Figure S3. Histogram presentation of GO ontology classification with 1306 genes that were downregulated in T.castaneum larvae after CBP knockdown. Figure S4. Epi-factor domains within the downregulated genes (1306) after CBP knockdown in T.castaneum larvae. Figure S5. KEGG pathway analysis. Figure S6. Correlation of gene expression levels of 20 selected genes by comparing both qPCR and RNA-seq data. Supporting Information S1. KEGG pathway analysis output. (DOCX 2490 kb) [file 12864_2017_4373_MOESM1_ESM.docx]

**Multiple functions of CREB-binding protein during postembryonic development: Identification of target genes**

*Amit Roy, Smitha George and Subba Reddy Palli**

Department of Entomology, College of Agriculture, University of Kentucky, Lexington, KY 40546, USA

**List of materials included**

**Table S1**: Sequences of primers used in the experiments.

**Figure S1**: Checking the knockdown efficiency in *T.castaneum* larvae and cDNA library preparation for RNA seq.

**Figure S2**: Normalization of RNA-seq data.

**Figure S3**: Histogram presentation of GO ontology classification with 1306 genes that were downregulated in *T.castaneum* larvae after CBP knockdown.

**Figure S4**: Epi-factor domains within the downregulated genes (1306) after CBP knockdown in *T.castaneum* larvae.

**Figure S5**: KEGG pathway analysis.

**Figure S6**: Correlation of gene expression levels of 20 selected genes by comparing both qPCR and RNA-seq data.

**Supporting Information S1:** KEGG pathway analysis output.

**Excel file S1**: Details of 1306 genes downregulated by CBP RNAi.

**Excel file S2**: Details of 52 genes identified after k-mer clustering.

**
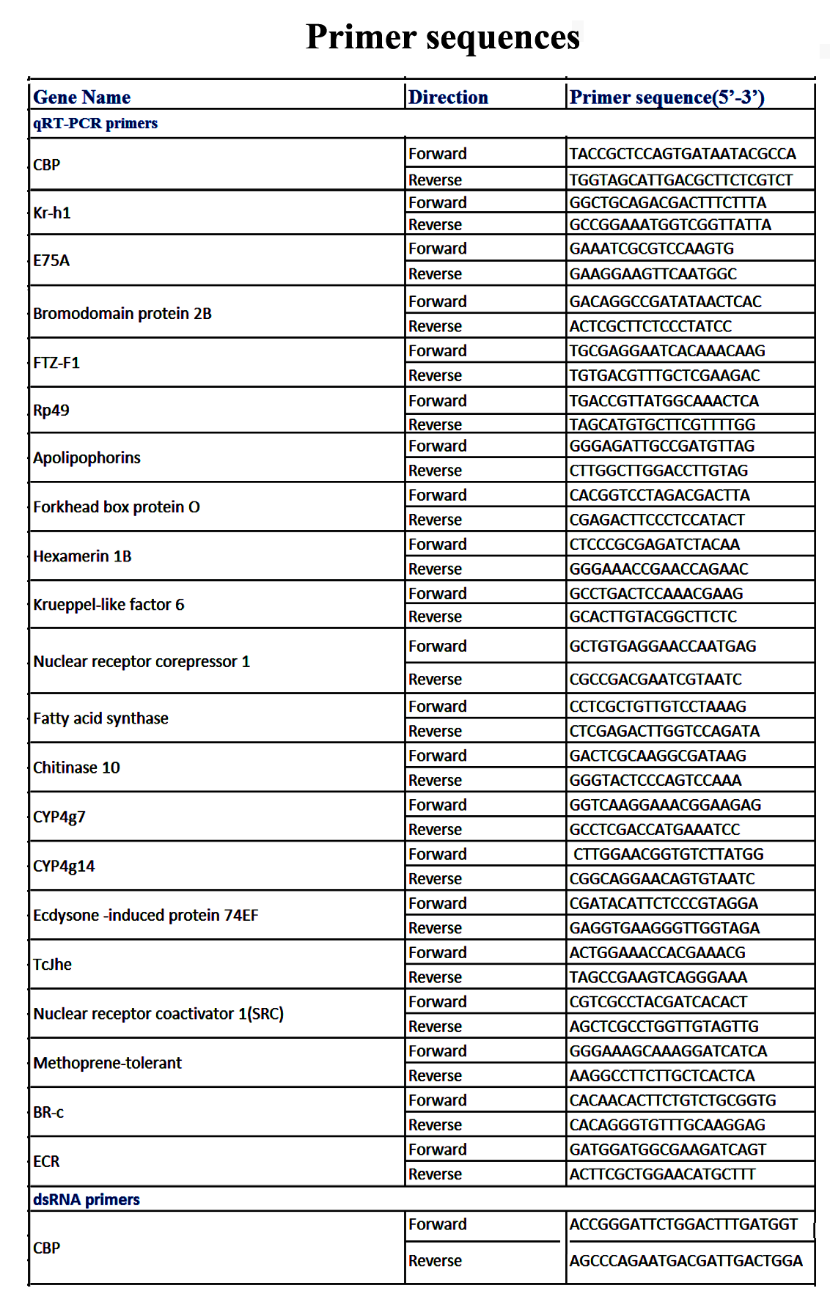
 Table S1: Sequences of primers used in the experiments.**

**Figure S1**

**
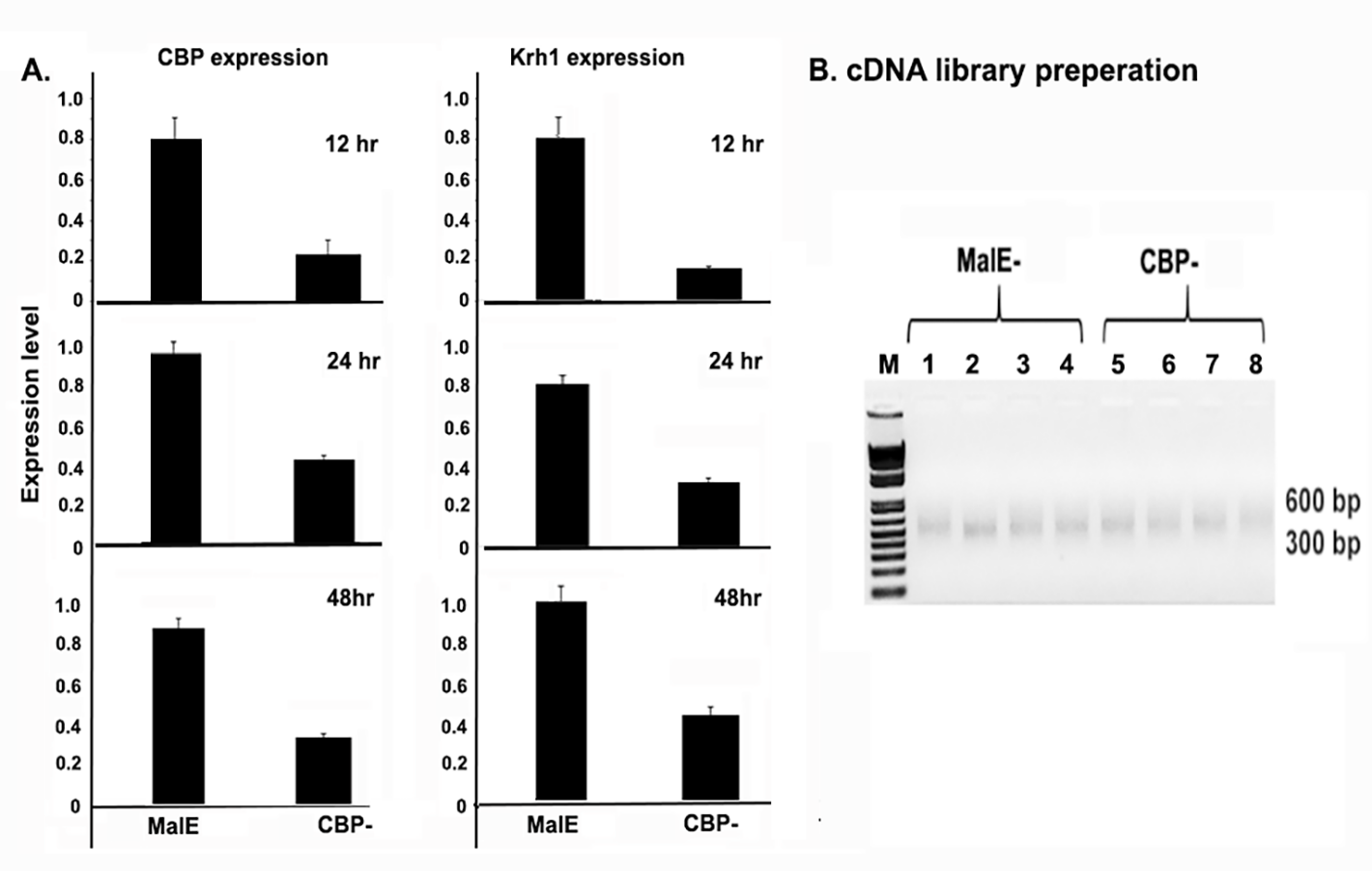
**

**Figure S1**: **Checking the knockdown efficiency in *T.castaneum* larvae and cDNA library preparation for RNA seq.** (A) qPCR based expression analysis of CBP and Kr-h1 gene expression after injection of dsmalE and dsCBP at different time points after injection. RNA samples from 12 hr time point are taken for downstream cDNA library preparation and sequencing. (B) The outcome of RNA-seq library preparation. 1.5% agarose gel showing the amplicon size of the prepared cDNA libraries after size selection.

**Figure S2**


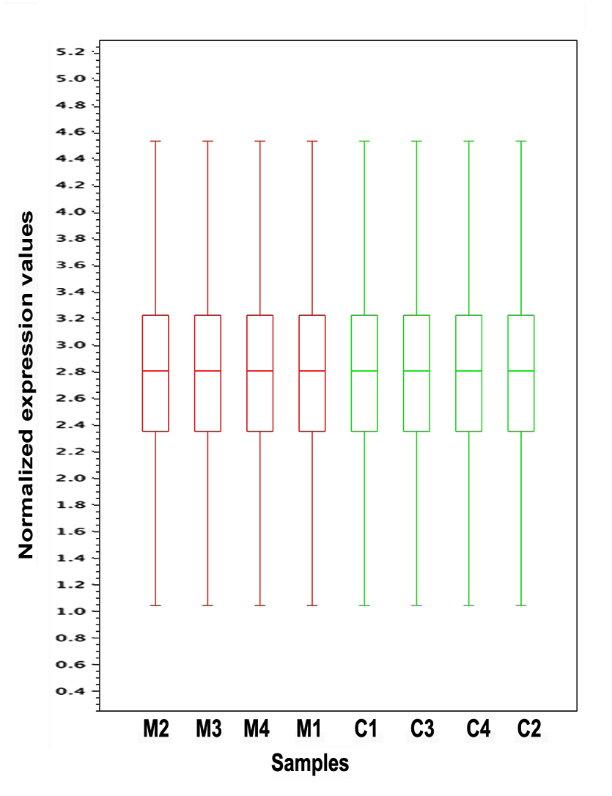


**Figure S2: Normalization of RNA-seq data.** Box-plot illustrating a high level of expression normalization in control (dsmalE injected) and CBP (dsCBP injected samples.

**Figure S3**

**
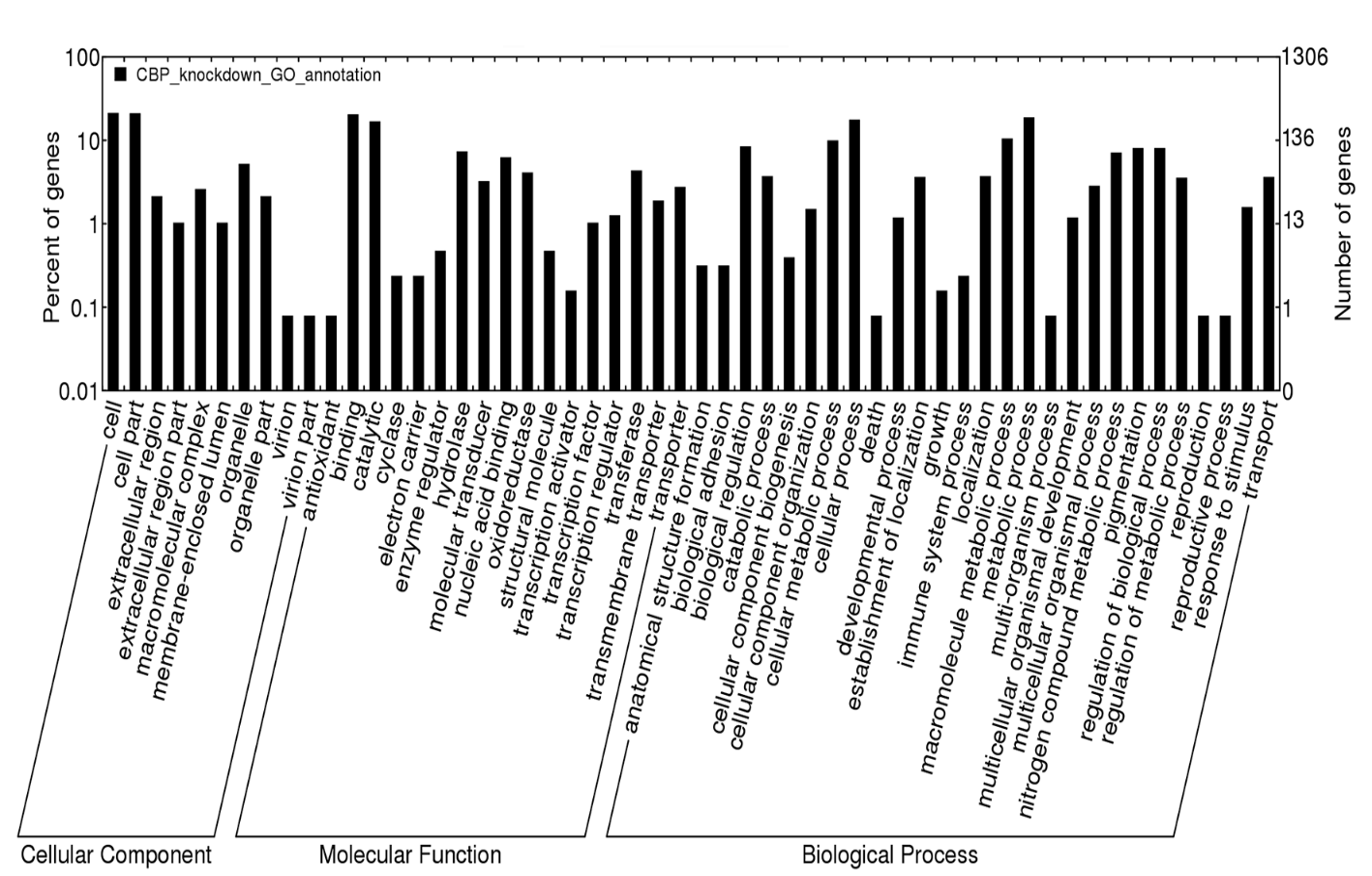
**

**Figure S3: Histogram presentation of GO ontology classification with 1306 genes that were downregulated in *T.castaneum* larvae after CBP knockdown**. Classification and functional distribution of the selected 1306 unigenes were represented according to the three major classifications of gene ontology: Biological Process, Molecular Function and Cellular Component.

**Figure S4**


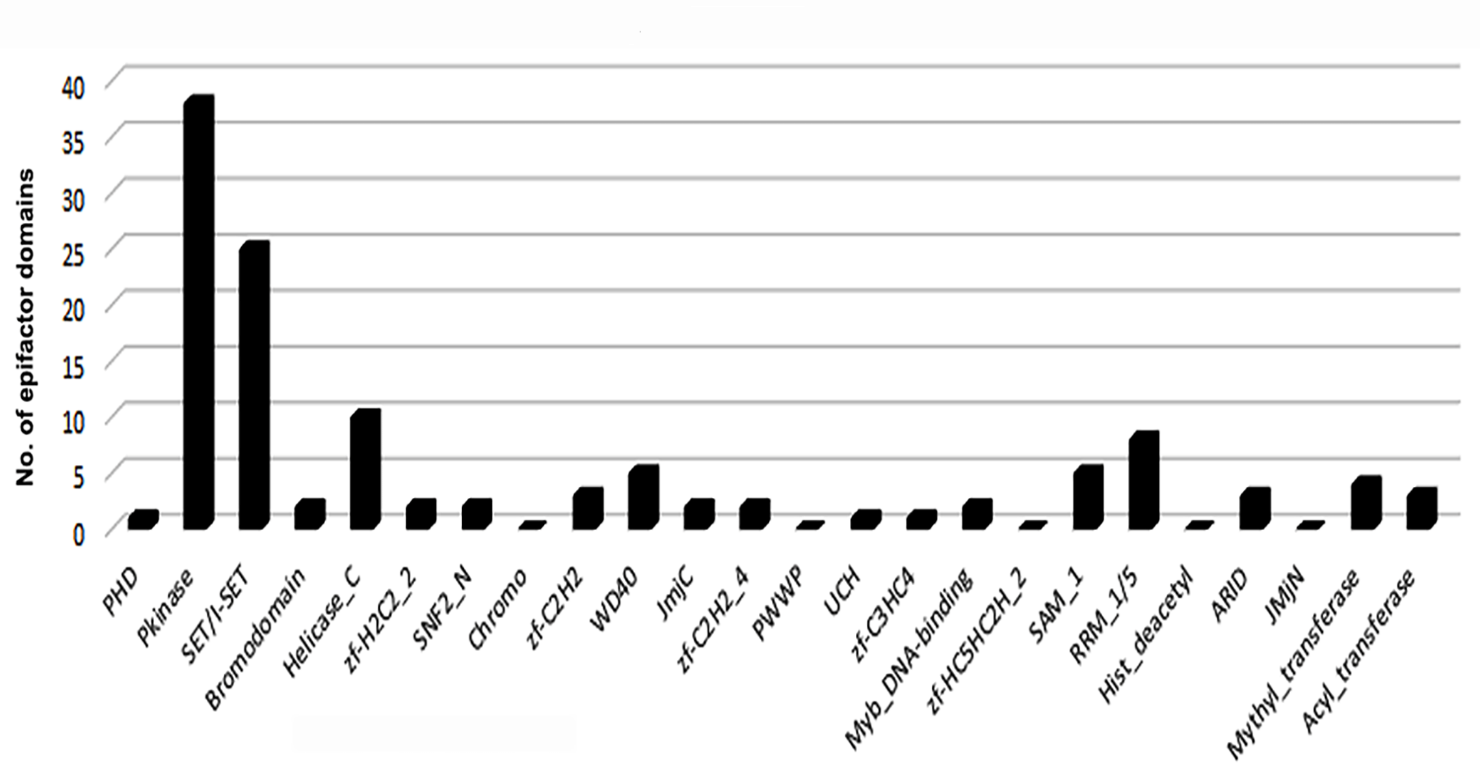


**Figure S4**: **Epi-factor domains within the downregulated genes (1306) after CBP knockdown in *T.cascaneum* larvae.** High number different epi-factor domains were observed in the target genes indicating their plausible role in epigenetic modulation in *T. castaenum*.

**Figure S5**


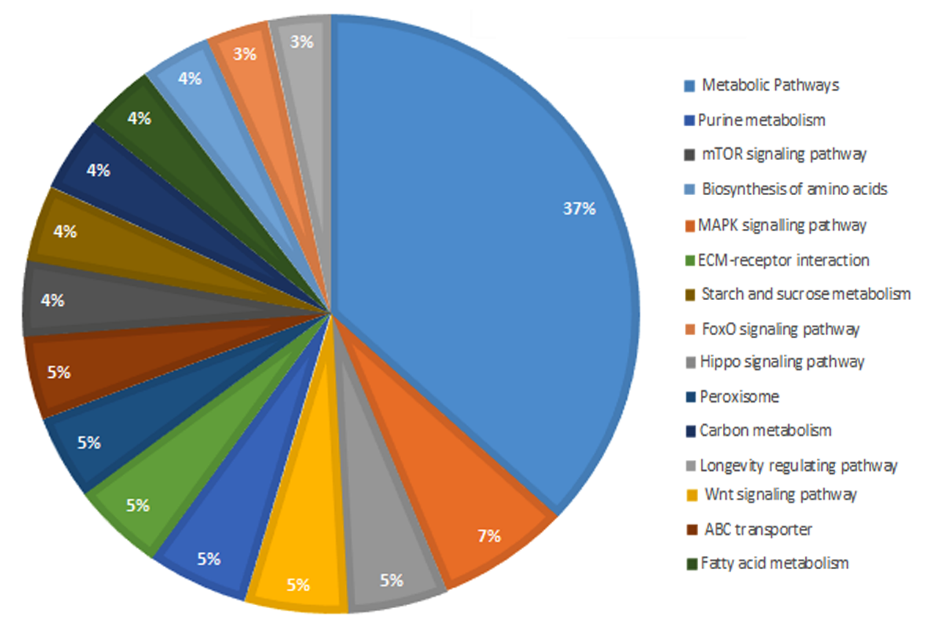


**Figure S5**: **KEGG pathway analysis.** Listed top 15 physiological processes affected by CBP knockdown in *T.castaneum* larvae.

**Figure S6**


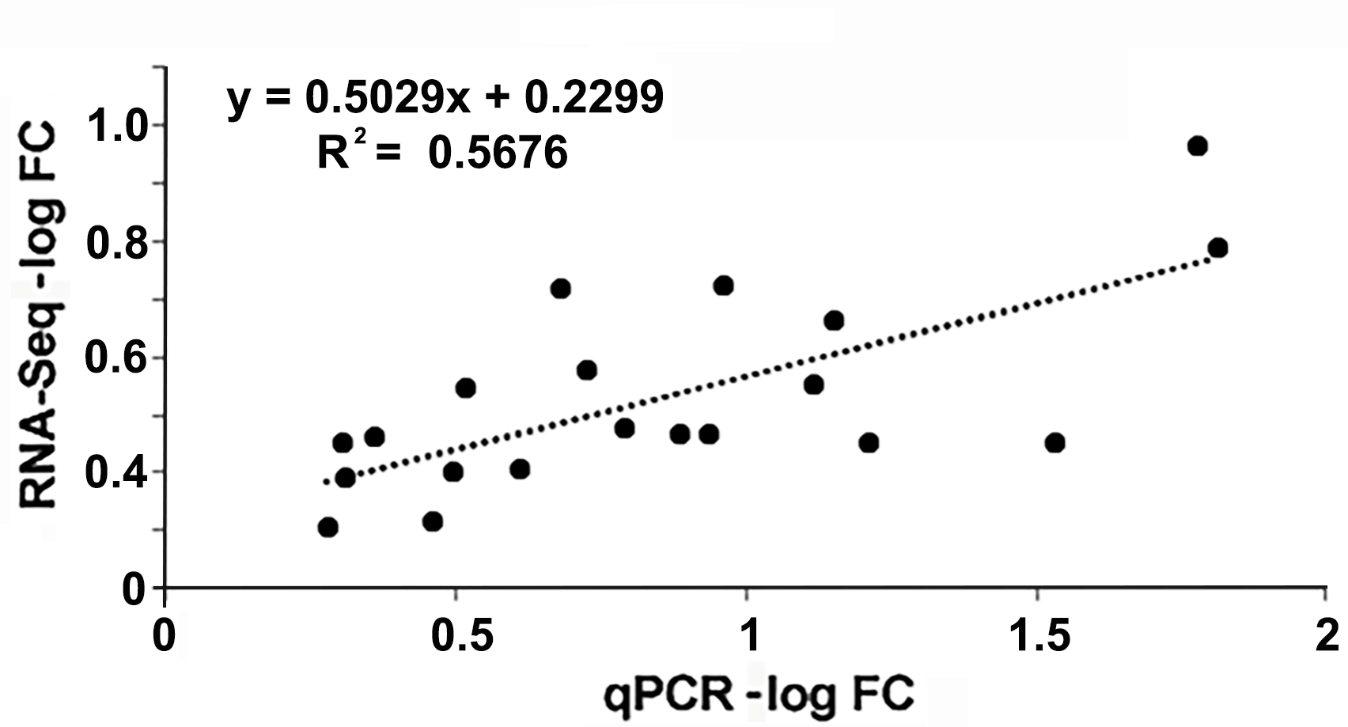


**Figure S6**: **Correlation of gene expression levels of 20 selected genes by comparing both qPCR and RNA-seq data.** Individual log fold changes obtained by qPCR and RNA-seq for each gene in the sample group.

**Supporting Information S1**

**KEGG pathway analysis output: Top 15 pathways affected by CBP RNAi**

List of genes affected by CBP RNAi from each of the pathways given below:

 **[tca01100 Metabolic pathways - Tribolium castaneum (red flour beetle)](http://www.kegg.jp/kegg-bin/show_pathway?148760529482229/tca01100.args) (**[**90**](javascript:display('tca01100'))**)**

[tca:100141596](http://www.kegg.jp/dbget-bin/www_bget?tca:100141596) glycoprotein-N-acetylgalactosamine 3-beta-galactosyltransferase 1

[tca:100142126](http://www.kegg.jp/dbget-bin/www_bget?tca:100142126) hypothetical protein

[tca:103312199](http://www.kegg.jp/dbget-bin/www_bget?tca:103312199) sialic acid synthase

[tca:103312225](http://www.kegg.jp/dbget-bin/www_bget?tca:103312225) adenosine deaminase CECR1-A

[tca:103312380](http://www.kegg.jp/dbget-bin/www_bget?tca:103312380) diacylglycerol kinase eta

[tca:641601](http://www.kegg.jp/dbget-bin/www_bget?tca:641601) Cht5; chitinase 5

[tca:652967](http://www.kegg.jp/dbget-bin/www_bget?tca:652967) Cht10; chitinase 10

[tca:654917](http://www.kegg.jp/dbget-bin/www_bget?tca:654917) alpha-1,3-mannosyl-glycoprotein 4-beta-N-acetylglucosaminyltransferase B

[tca:655213](http://www.kegg.jp/dbget-bin/www_bget?tca:655213) UDP-glucose 4-epimerase

[tca:655392](http://www.kegg.jp/dbget-bin/www_bget?tca:655392) protein henna

[tca:655418](http://www.kegg.jp/dbget-bin/www_bget?tca:655418) sphingomyelin phosphodiesterase

[tca:655432](http://www.kegg.jp/dbget-bin/www_bget?tca:655432) purine nucleoside phosphorylase

[tca:655549](http://www.kegg.jp/dbget-bin/www_bget?tca:655549) nitric oxide synthase, salivary gland

[tca:655758](http://www.kegg.jp/dbget-bin/www_bget?tca:655758) tryptophan 5-hydroxylase 1

[tca:656036](http://www.kegg.jp/dbget-bin/www_bget?tca:656036) glycerol-3-phosphate acyltransferase 1, mitochondrial

[tca:656073](http://www.kegg.jp/dbget-bin/www_bget?tca:656073) chondroitin sulfate synthase 1

[tca:656134](http://www.kegg.jp/dbget-bin/www_bget?tca:656134) argininosuccinate lyase

[tca:656241](http://www.kegg.jp/dbget-bin/www_bget?tca:656241) bifunctional purine biosynthesis protein PURH

[tca:656545](http://www.kegg.jp/dbget-bin/www_bget?tca:656545) amidophosphoribosyltransferase

[tca:656564](http://www.kegg.jp/dbget-bin/www_bget?tca:656564) alanine--glyoxylate aminotransferase 2-like

[tca:656621](http://www.kegg.jp/dbget-bin/www_bget?tca:656621) xanthine dehydrogenase

[tca:656820](http://www.kegg.jp/dbget-bin/www_bget?tca:656820) C-1-tetrahydrofolate synthase, cytoplasmic

[tca:656825](http://www.kegg.jp/dbget-bin/www_bget?tca:656825) peroxiredoxin-6

[tca:656849](http://www.kegg.jp/dbget-bin/www_bget?tca:656849) sarcosine dehydrogenase, mitochondrial

[tca:657046](http://www.kegg.jp/dbget-bin/www_bget?tca:657046) serine--pyruvate aminotransferase, mitochondrial

[tca:657084](http://www.kegg.jp/dbget-bin/www_bget?tca:657084) phospholipase B1, membrane-associated

[tca:657239](http://www.kegg.jp/dbget-bin/www_bget?tca:657239) aminopeptidase N

[tca:657312](http://www.kegg.jp/dbget-bin/www_bget?tca:657312) aminopeptidase N

[tca:657315](http://www.kegg.jp/dbget-bin/www_bget?tca:657315) neutral ceramidase

[tca:657406](http://www.kegg.jp/dbget-bin/www_bget?tca:657406) glycogen phosphorylase

[tca:657468](http://www.kegg.jp/dbget-bin/www_bget?tca:657468) neutral ceramidase-like

[tca:657660](http://www.kegg.jp/dbget-bin/www_bget?tca:657660) DNA polymerase epsilon catalytic subunit A

[tca:657686](http://www.kegg.jp/dbget-bin/www_bget?tca:657686) NADP-dependent malic enzyme

[tca:657796](http://www.kegg.jp/dbget-bin/www_bget?tca:657796) inositol-trisphosphate 3-kinase A

[tca:657862](http://www.kegg.jp/dbget-bin/www_bget?tca:657862) prolyl 4-hydroxylase subunit alpha-2

[tca:657963](http://www.kegg.jp/dbget-bin/www_bget?tca:657963) methylcrotonoyl-CoA carboxylase subunit alpha, mitochondrial

[tca:658024](http://www.kegg.jp/dbget-bin/www_bget?tca:658024) fatty acid synthase

[tca:658173](http://www.kegg.jp/dbget-bin/www_bget?tca:658173) alpha-1,6-mannosyl-glycoprotein 2-beta-N-acetylglucosaminyltransferase

[tca:658249](http://www.kegg.jp/dbget-bin/www_bget?tca:658249) Nag2; beta-N-acetylglucosaminidase NAG2

[tca:658327](http://www.kegg.jp/dbget-bin/www_bget?tca:658327) ATP-dependent 6-phosphofructokinase

[tca:658362](http://www.kegg.jp/dbget-bin/www_bget?tca:658362) acetyl-CoA carboxylase

[tca:658558](http://www.kegg.jp/dbget-bin/www_bget?tca:658558) aldose reductase

[tca:658584](http://www.kegg.jp/dbget-bin/www_bget?tca:658584) glutamate synthase 1 [NADH], chloroplastic

[tca:658613](http://www.kegg.jp/dbget-bin/www_bget?tca:658613) glycine dehydrogenase (decarboxylating), mitochondrial

[tca:658917](http://www.kegg.jp/dbget-bin/www_bget?tca:658917) delta-1-pyrroline-5-carboxylate synthase

[tca:658942](http://www.kegg.jp/dbget-bin/www_bget?tca:658942) NAD(P) transhydrogenase, mitochondrial

[tca:658959](http://www.kegg.jp/dbget-bin/www_bget?tca:658959) putative polypeptide N-acetylgalactosaminyltransferase 9

[tca:659029](http://www.kegg.jp/dbget-bin/www_bget?tca:659029) long-chain-fatty-acid--CoA ligase 5

[tca:659179](http://www.kegg.jp/dbget-bin/www_bget?tca:659179) fatty acid synthase

[tca:659253](http://www.kegg.jp/dbget-bin/www_bget?tca:659253) L-threonine ammonia-lyase

[tca:659356](http://www.kegg.jp/dbget-bin/www_bget?tca:659356) alpha,alpha-trehalose-phosphate synthase [UDP-forming]

[tca:659675](http://www.kegg.jp/dbget-bin/www_bget?tca:659675) DNA primase small subunit

[tca:659687](http://www.kegg.jp/dbget-bin/www_bget?tca:659687) protein O-mannosyltransferase 1

[tca:660178](http://www.kegg.jp/dbget-bin/www_bget?tca:660178) fatty acid synthase

[tca:660290](http://www.kegg.jp/dbget-bin/www_bget?tca:660290) UDP-glucuronosyltransferase 1-7

[tca:660388](http://www.kegg.jp/dbget-bin/www_bget?tca:660388) alpha-aminoadipic semialdehyde synthase, mitochondrial

[tca:660529](http://www.kegg.jp/dbget-bin/www_bget?tca:660529) UDP-N-acetylglucosamine transferase subunit ALG14 homolog

[tca:660733](http://www.kegg.jp/dbget-bin/www_bget?tca:660733) L-xylulose reductase

[tca:660813](http://www.kegg.jp/dbget-bin/www_bget?tca:660813) NADH-ubiquinone oxidoreductase 49 kDa subunit

[tca:660841](http://www.kegg.jp/dbget-bin/www_bget?tca:660841) L-xylulose reductase

[tca:660846](http://www.kegg.jp/dbget-bin/www_bget?tca:660846) UDP-glucuronosyltransferase 2C1

[tca:660900](http://www.kegg.jp/dbget-bin/www_bget?tca:660900) CAD protein

[tca:660961](http://www.kegg.jp/dbget-bin/www_bget?tca:660961) cytosolic non-specific dipeptidase

[tca:661002](http://www.kegg.jp/dbget-bin/www_bget?tca:661002) lactase-phlorizin hydrolase-like

[tca:661138](http://www.kegg.jp/dbget-bin/www_bget?tca:661138) eye-specific diacylglycerol kinase

[tca:661312](http://www.kegg.jp/dbget-bin/www_bget?tca:661312) 1-phosphatidylinositol 4,5-bisphosphate phosphodiesterase epsilon-1

[tca:661428](http://www.kegg.jp/dbget-bin/www_bget?tca:661428) Cht8; chitinase 8

[tca:661503](http://www.kegg.jp/dbget-bin/www_bget?tca:661503) propionyl-CoA carboxylase beta chain, mitochondrial

[tca:661511](http://www.kegg.jp/dbget-bin/www_bget?tca:661511) UDP-glucuronosyltransferase 1-9

[tca:661583](http://www.kegg.jp/dbget-bin/www_bget?tca:661583) LanA; laminin subunit alpha

[tca:662050](http://www.kegg.jp/dbget-bin/www_bget?tca:662050) glycogen [starch] synthase

[tca:662176](http://www.kegg.jp/dbget-bin/www_bget?tca:662176) homocysteine S-methyltransferase

[tca:662431](http://www.kegg.jp/dbget-bin/www_bget?tca:662431) branched-chain-amino-acid aminotransferase, cytosolic

[tca:662526](http://www.kegg.jp/dbget-bin/www_bget?tca:662526) glycogen debranching enzyme

[tca:662767](http://www.kegg.jp/dbget-bin/www_bget?tca:662767) N-acetylgalactosaminyltransferase 7

[tca:662782](http://www.kegg.jp/dbget-bin/www_bget?tca:662782) trehalase

[tca:662899](http://www.kegg.jp/dbget-bin/www_bget?tca:662899) fatty acid synthase

[tca:663218](http://www.kegg.jp/dbget-bin/www_bget?tca:663218) D-3-phosphoglycerate dehydrogenase

[tca:663325](http://www.kegg.jp/dbget-bin/www_bget?tca:663325) probable phosphoserine aminotransferase

[tca:663399](http://www.kegg.jp/dbget-bin/www_bget?tca:663399) fructose-1,6-bisphosphatase isozyme 2-like

[tca:663547](http://www.kegg.jp/dbget-bin/www_bget?tca:663547) PCCA; propionyl-CoA carboxylase alpha chain, mitochondrial

[tca:663954](http://www.kegg.jp/dbget-bin/www_bget?tca:663954) alpha amylase

[tca:664022](http://www.kegg.jp/dbget-bin/www_bget?tca:664022) alpha-amylase

[tca:664278](http://www.kegg.jp/dbget-bin/www_bget?tca:664278) gephyrin

[tca:664385](http://www.kegg.jp/dbget-bin/www_bget?tca:664385) alpha-amylase

[tca:664389](http://www.kegg.jp/dbget-bin/www_bget?tca:664389) alpha-amylase-like

[tca:664392](http://www.kegg.jp/dbget-bin/www_bget?tca:664392) alpha-amylase

[tca:664486](http://www.kegg.jp/dbget-bin/www_bget?tca:664486) phosphatidate phosphatase LPIN3

[tca:664509](http://www.kegg.jp/dbget-bin/www_bget?tca:664509) enolase-phosphatase E1

[tca:664593](http://www.kegg.jp/dbget-bin/www_bget?tca:664593) histidine decarboxylase

 **[tca04013 MAPK signaling pathway - fly - Tribolium castaneum (red flour beetle)](http://www.kegg.jp/kegg-bin/show_pathway?148760529482229/tca04013.args) (**[**17**](javascript:display('tca04013'))**)**

[tca:100141824](http://www.kegg.jp/dbget-bin/www_bget?tca:100141824) Krn; uncharacterized LOC100141824

[tca:103312877](http://www.kegg.jp/dbget-bin/www_bget?tca:103312877) Nasrat; uncharacterized LOC103312877

[tca:103313241](http://www.kegg.jp/dbget-bin/www_bget?tca:103313241) uncharacterized LOC103313241

[tca:655394](http://www.kegg.jp/dbget-bin/www_bget?tca:655394) ras GTPase-activating protein 1

[tca:656565](http://www.kegg.jp/dbget-bin/www_bget?tca:656565) tyrosine-protein kinase Src64B

[tca:659260](http://www.kegg.jp/dbget-bin/www_bget?tca:659260) thickveins; bone morphogenetic protein receptor type-1B

[tca:659567](http://www.kegg.jp/dbget-bin/www_bget?tca:659567) sev; proto-oncogene tyrosine-protein kinase ROS

[tca:660037](http://www.kegg.jp/dbget-bin/www_bget?tca:660037) DER; epidermal growth factor receptor

[tca:660060](http://www.kegg.jp/dbget-bin/www_bget?tca:660060) lz; lozenge

[tca:660328](http://www.kegg.jp/dbget-bin/www_bget?tca:660328) pros; homeobox protein prospero

[tca:660448](http://www.kegg.jp/dbget-bin/www_bget?tca:660448) myocyte-specific enhancer factor 2

[tca:661159](http://www.kegg.jp/dbget-bin/www_bget?tca:661159) Omb; optomotor-blind-like

[tca:661670](http://www.kegg.jp/dbget-bin/www_bget?tca:661670) hypothetical protein

[tca:661922](http://www.kegg.jp/dbget-bin/www_bget?tca:661922) sprouty; protein sprouty

[tca:662162](http://www.kegg.jp/dbget-bin/www_bget?tca:662162) Peb; pebbled

[tca:664119](http://www.kegg.jp/dbget-bin/www_bget?tca:664119) tumor necrosis factor receptor superfamily member wengen

[tca:664510](http://www.kegg.jp/dbget-bin/www_bget?tca:664510) mitogen-activated protein kinase kinase kinase 11

** [tca00230 Purine metabolism - Tribolium castaneum (red flour beetle)](http://www.kegg.jp/kegg-bin/show_pathway?148760529482229/tca00230.args) (**[**13**](javascript:display('tca00230'))**)**

[tca:103312225](http://www.kegg.jp/dbget-bin/www_bget?tca:103312225) adenosine deaminase CECR1-A

[tca:107397949](http://www.kegg.jp/dbget-bin/www_bget?tca:107397949) adenylate cyclase type 8-like

[tca:655273](http://www.kegg.jp/dbget-bin/www_bget?tca:655273) adenylate cyclase type 8

[tca:655432](http://www.kegg.jp/dbget-bin/www_bget?tca:655432) purine nucleoside phosphorylase

[tca:656241](http://www.kegg.jp/dbget-bin/www_bget?tca:656241) bifunctional purine biosynthesis protein PURH

[tca:656545](http://www.kegg.jp/dbget-bin/www_bget?tca:656545) amidophosphoribosyltransferase

[tca:656621](http://www.kegg.jp/dbget-bin/www_bget?tca:656621) xanthine dehydrogenase

[tca:657274](http://www.kegg.jp/dbget-bin/www_bget?tca:657274) retained; high affinity cAMP-specific and IBMX-insensitive 3',5'-cyclic phosphodiesterase 8A

[tca:657660](http://www.kegg.jp/dbget-bin/www_bget?tca:657660) DNA polymerase epsilon catalytic subunit A

[tca:658906](http://www.kegg.jp/dbget-bin/www_bget?tca:658906) high affinity cGMP-specific 3',5'-cyclic phosphodiesterase 9A

[tca:659510](http://www.kegg.jp/dbget-bin/www_bget?tca:659510) probable 3',5'-cyclic phosphodiesterase pde-5

[tca:659675](http://www.kegg.jp/dbget-bin/www_bget?tca:659675) DNA primase small subunit

[tca:661438](http://www.kegg.jp/dbget-bin/www_bget?tca:661438) rut; Ca(2+)/calmodulin-responsive adenylate cyclase

 **[tca04310 Wnt signaling pathway - Tribolium castaneum (red flour beetle)](http://www.kegg.jp/kegg-bin/show_pathway?148760529482229/tca04310.args) (**[**13**](javascript:display('tca04310'))**)**

[tca:103314170](http://www.kegg.jp/dbget-bin/www_bget?tca:103314170) bambi; BMP and activin membrane-bound inhibitor homolog

[tca:655266](http://www.kegg.jp/dbget-bin/www_bget?tca:655266) calcium/calmodulin-dependent protein kinase type II alpha chain

[tca:655873](http://www.kegg.jp/dbget-bin/www_bget?tca:655873) Axn; axis inhibition protein

[tca:655955](http://www.kegg.jp/dbget-bin/www_bget?tca:655955) Fz4; frizzled 4

[tca:656250](http://www.kegg.jp/dbget-bin/www_bget?tca:656250) protein kinase C, brain isozyme-like

[tca:656499](http://www.kegg.jp/dbget-bin/www_bget?tca:656499) Fz2; frizzled 2

[tca:656598](http://www.kegg.jp/dbget-bin/www_bget?tca:656598) Wnt10; protein Wnt-10a

[tca:656854](http://www.kegg.jp/dbget-bin/www_bget?tca:656854) division abnormally delayed protein

[tca:657726](http://www.kegg.jp/dbget-bin/www_bget?tca:657726) Wnt11; protein Wnt-11b-1

[tca:659674](http://www.kegg.jp/dbget-bin/www_bget?tca:659674) serine/threonine-protein kinase NLK

[tca:661080](http://www.kegg.jp/dbget-bin/www_bget?tca:661080) uncharacterized LOC661080

[tca:661337](http://www.kegg.jp/dbget-bin/www_bget?tca:661337) protein prickle-like

[tca:661936](http://www.kegg.jp/dbget-bin/www_bget?tca:661936) Wnt7; protein Wnt-7b

 **[tca04391 Hippo signaling pathway - fly - Tribolium castaneum (red flour beetle)](http://www.kegg.jp/kegg-bin/show_pathway?148760529482229/tca04391.args) (**[**13**](javascript:display('tca04391'))**)**

[tca:100141824](http://www.kegg.jp/dbget-bin/www_bget?tca:100141824) Krn; uncharacterized LOC100141824

[tca:103313241](http://www.kegg.jp/dbget-bin/www_bget?tca:103313241) uncharacterized LOC103313241

[tca:656854](http://www.kegg.jp/dbget-bin/www_bget?tca:656854) division abnormally delayed protein

[tca:657914](http://www.kegg.jp/dbget-bin/www_bget?tca:657914) Myo20; myosin 20

[tca:658147](http://www.kegg.jp/dbget-bin/www_bget?tca:658147) hemicentin-2

[tca:658922](http://www.kegg.jp/dbget-bin/www_bget?tca:658922) Sd; scalloped

[tca:659267](http://www.kegg.jp/dbget-bin/www_bget?tca:659267) protein expanded

[tca:659713](http://www.kegg.jp/dbget-bin/www_bget?tca:659713) FAT; cadherin-related tumor suppressor

[tca:660913](http://www.kegg.jp/dbget-bin/www_bget?tca:660913) LIX1-like protein

[tca:661284](http://www.kegg.jp/dbget-bin/www_bget?tca:661284) partitioning defective 3 homolog B

[tca:662006](http://www.kegg.jp/dbget-bin/www_bget?tca:662006) dachsous; protein dachsous

[tca:663662](http://www.kegg.jp/dbget-bin/www_bget?tca:663662) protein kibra

[tca:664119](http://www.kegg.jp/dbget-bin/www_bget?tca:664119) tumor necrosis factor receptor superfamily member wengen

 **[tca04512 ECM-receptor interaction - Tribolium castaneum (red flour beetle)](http://www.kegg.jp/kegg-bin/show_pathway?148760529482229/tca04512.args) (**[**12**](javascript:display('tca04512'))**)**

[tca:100141619](http://www.kegg.jp/dbget-bin/www_bget?tca:100141619) collagen alpha-3(IX) chain

[tca:100141763](http://www.kegg.jp/dbget-bin/www_bget?tca:100141763) agrin

[tca:103313111](http://www.kegg.jp/dbget-bin/www_bget?tca:103313111) collagen alpha-2(IV) chain

[tca:657051](http://www.kegg.jp/dbget-bin/www_bget?tca:657051) laminin subunit gamma-1

[tca:658046](http://www.kegg.jp/dbget-bin/www_bget?tca:658046) laminin subunit alpha-1

[tca:659601](http://www.kegg.jp/dbget-bin/www_bget?tca:659601) integrin alpha-PS1

[tca:660112](http://www.kegg.jp/dbget-bin/www_bget?tca:660112) fibril-forming collagen alpha chain

[tca:661297](http://www.kegg.jp/dbget-bin/www_bget?tca:661297) laminin subunit beta-1

[tca:661583](http://www.kegg.jp/dbget-bin/www_bget?tca:661583) LanA; laminin subunit alpha

[tca:662246](http://www.kegg.jp/dbget-bin/www_bget?tca:662246) synaptic vesicle glycoprotein 2B

[tca:663310](http://www.kegg.jp/dbget-bin/www_bget?tca:663310) collagen alpha-2(IV) chain

[tca:663372](http://www.kegg.jp/dbget-bin/www_bget?tca:663372) dystroglycan

 **[tca02010 ABC transporters - Tribolium castaneum (red flour beetle)](http://www.kegg.jp/kegg-bin/show_pathway?148760529482229/tca02010.args) (**[**11**](javascript:display('tca02010'))**)**

[tca:100142393](http://www.kegg.jp/dbget-bin/www_bget?tca:100142393) cystic fibrosis transmembrane conductance regulator

[tca:100142464](http://www.kegg.jp/dbget-bin/www_bget?tca:100142464) multidrug resistance-associated protein 4-like

[tca:107398636](http://www.kegg.jp/dbget-bin/www_bget?tca:107398636) multidrug resistance-associated protein 4-like

[tca:656043](http://www.kegg.jp/dbget-bin/www_bget?tca:656043) ATP-binding cassette sub-family A member 1-like

[tca:657736](http://www.kegg.jp/dbget-bin/www_bget?tca:657736) ATP-binding cassette sub-family A member 2

[tca:658871](http://www.kegg.jp/dbget-bin/www_bget?tca:658871) probable multidrug resistance-associated protein lethal(2)03659

[tca:658981](http://www.kegg.jp/dbget-bin/www_bget?tca:658981) probable multidrug resistance-associated protein lethal(2)03659

[tca:660313](http://www.kegg.jp/dbget-bin/www_bget?tca:660313) ATP-binding cassette sub-family D member 3

[tca:660350](http://www.kegg.jp/dbget-bin/www_bget?tca:660350) ATP-binding cassette sub-family G member 1

[tca:660357](http://www.kegg.jp/dbget-bin/www_bget?tca:660357) probable multidrug resistance-associated protein lethal(2)03659

[tca:660659](http://www.kegg.jp/dbget-bin/www_bget?tca:660659) multidrug resistance-associated protein 4

 **[tca04146 Peroxisome - Tribolium castaneum (red flour beetle)](http://www.kegg.jp/kegg-bin/show_pathway?148760529482229/tca04146.args) (**[**11**](javascript:display('tca04146'))**)**

[tca:100141869](http://www.kegg.jp/dbget-bin/www_bget?tca:100141869) putative fatty acyl-CoA reductase CG5065

[tca:656115](http://www.kegg.jp/dbget-bin/www_bget?tca:656115) putative fatty acyl-CoA reductase CG5065

[tca:656621](http://www.kegg.jp/dbget-bin/www_bget?tca:656621) xanthine dehydrogenase

[tca:656776](http://www.kegg.jp/dbget-bin/www_bget?tca:656776) putative fatty acyl-CoA reductase CG5065

[tca:657046](http://www.kegg.jp/dbget-bin/www_bget?tca:657046) serine--pyruvate aminotransferase, mitochondrial

[tca:659029](http://www.kegg.jp/dbget-bin/www_bget?tca:659029) long-chain-fatty-acid--CoA ligase 5

[tca:659998](http://www.kegg.jp/dbget-bin/www_bget?tca:659998) putative fatty acyl-CoA reductase CG5065

[tca:660187](http://www.kegg.jp/dbget-bin/www_bget?tca:660187) putative fatty acyl-CoA reductase CG5065

[tca:660313](http://www.kegg.jp/dbget-bin/www_bget?tca:660313) ATP-binding cassette sub-family D member 3

[tca:662263](http://www.kegg.jp/dbget-bin/www_bget?tca:662263) putative fatty acyl-CoA reductase CG8306

[tca:663534](http://www.kegg.jp/dbget-bin/www_bget?tca:663534) putative fatty acyl-CoA reductase CG5065

 **[tca00500 Starch and sucrose metabolism - Tribolium castaneum (red flour beetle)](http://www.kegg.jp/kegg-bin/show_pathway?148760529482229/tca00500.args) (**[**10**](javascript:display('tca00500'))**)**

[tca:657406](http://www.kegg.jp/dbget-bin/www_bget?tca:657406) glycogen phosphorylase

[tca:659356](http://www.kegg.jp/dbget-bin/www_bget?tca:659356) alpha,alpha-trehalose-phosphate synthase [UDP-forming]

[tca:662050](http://www.kegg.jp/dbget-bin/www_bget?tca:662050) glycogen [starch] synthase

[tca:662526](http://www.kegg.jp/dbget-bin/www_bget?tca:662526) glycogen debranching enzyme

[tca:662782](http://www.kegg.jp/dbget-bin/www_bget?tca:662782) trehalase

[tca:663954](http://www.kegg.jp/dbget-bin/www_bget?tca:663954) alpha amylase

[tca:664022](http://www.kegg.jp/dbget-bin/www_bget?tca:664022) alpha-amylase

[tca:664385](http://www.kegg.jp/dbget-bin/www_bget?tca:664385) alpha-amylase

[tca:664389](http://www.kegg.jp/dbget-bin/www_bget?tca:664389) alpha-amylase-like

[tca:664392](http://www.kegg.jp/dbget-bin/www_bget?tca:664392) alpha-amylase

** [tca01200 Carbon metabolism - Tribolium castaneum (red flour beetle)](http://www.kegg.jp/kegg-bin/show_pathway?148760529482229/tca01200.args) (**[**10**](javascript:display('tca01200'))**)**

[tca:657046](http://www.kegg.jp/dbget-bin/www_bget?tca:657046) serine--pyruvate aminotransferase, mitochondrial

[tca:657686](http://www.kegg.jp/dbget-bin/www_bget?tca:657686) NADP-dependent malic enzyme

[tca:658327](http://www.kegg.jp/dbget-bin/www_bget?tca:658327) ATP-dependent 6-phosphofructokinase

[tca:658613](http://www.kegg.jp/dbget-bin/www_bget?tca:658613) glycine dehydrogenase (decarboxylating), mitochondrial

[tca:659253](http://www.kegg.jp/dbget-bin/www_bget?tca:659253) L-threonine ammonia-lyase

[tca:661503](http://www.kegg.jp/dbget-bin/www_bget?tca:661503) propionyl-CoA carboxylase beta chain, mitochondrial

[tca:663218](http://www.kegg.jp/dbget-bin/www_bget?tca:663218) D-3-phosphoglycerate dehydrogenase

[tca:663325](http://www.kegg.jp/dbget-bin/www_bget?tca:663325) probable phosphoserine aminotransferase

[tca:663399](http://www.kegg.jp/dbget-bin/www_bget?tca:663399) fructose-1,6-bisphosphatase isozyme 2-like

[tca:663547](http://www.kegg.jp/dbget-bin/www_bget?tca:663547) PCCA; propionyl-CoA carboxylase alpha chain, mitochondrial

** [tca04150 mTOR signaling pathway - Tribolium castaneum (red flour beetle)](http://www.kegg.jp/kegg-bin/show_pathway?148760529482229/tca04150.args) (**[**10**](javascript:display('tca04150'))**)**

[tca:100142381](http://www.kegg.jp/dbget-bin/www_bget?tca:100142381) insulin-like peptide

[tca:655955](http://www.kegg.jp/dbget-bin/www_bget?tca:655955) Fz4; frizzled 4

[tca:656250](http://www.kegg.jp/dbget-bin/www_bget?tca:656250) protein kinase C, brain isozyme-like

[tca:656499](http://www.kegg.jp/dbget-bin/www_bget?tca:656499) Fz2; frizzled 2

[tca:656598](http://www.kegg.jp/dbget-bin/www_bget?tca:656598) Wnt10; protein Wnt-10a

[tca:657726](http://www.kegg.jp/dbget-bin/www_bget?tca:657726) Wnt11; protein Wnt-11b-1

[tca:660829](http://www.kegg.jp/dbget-bin/www_bget?tca:660829) sodium-coupled neutral amino acid transporter 9-like

[tca:661524](http://www.kegg.jp/dbget-bin/www_bget?tca:661524) InR; insulin-like receptor

[tca:661936](http://www.kegg.jp/dbget-bin/www_bget?tca:661936) Wnt7; protein Wnt-7b

[tca:664271](http://www.kegg.jp/dbget-bin/www_bget?tca:664271) ilr2; insulin-like receptor

 **[tca01212 Fatty acid metabolism - Tribolium castaneum (red flour beetle)](http://www.kegg.jp/kegg-bin/show_pathway?148760529482229/tca01212.args) (**[**9**](javascript:display('tca01212'))**)**

[tca:103314796](http://www.kegg.jp/dbget-bin/www_bget?tca:103314796) acyl-CoA Delta(11) desaturase

[tca:656186](http://www.kegg.jp/dbget-bin/www_bget?tca:656186) very-long-chain (3R)-3-hydroxyacyl-CoA dehydratase hpo-8

[tca:656478](http://www.kegg.jp/dbget-bin/www_bget?tca:656478) acyl-CoA Delta(11) desaturase-like

[tca:658024](http://www.kegg.jp/dbget-bin/www_bget?tca:658024) fatty acid synthase

[tca:658362](http://www.kegg.jp/dbget-bin/www_bget?tca:658362) acetyl-CoA carboxylase

[tca:659029](http://www.kegg.jp/dbget-bin/www_bget?tca:659029) long-chain-fatty-acid--CoA ligase 5

[tca:659179](http://www.kegg.jp/dbget-bin/www_bget?tca:659179) fatty acid synthase

[tca:660178](http://www.kegg.jp/dbget-bin/www_bget?tca:660178) fatty acid synthase

[tca:662899](http://www.kegg.jp/dbget-bin/www_bget?tca:662899) fatty acid synthase

 **[tca01230 Biosynthesis of amino acids - Tribolium castaneum (red flour beetle)](http://www.kegg.jp/kegg-bin/show_pathway?148760529482229/tca01230.args) (**[**9**](javascript:display('tca01230'))**)**

[**tca:655392**](http://www.kegg.jp/dbget-bin/www_bget?tca:655392) **protein henna**

[tca:656134](http://www.kegg.jp/dbget-bin/www_bget?tca:656134) argininosuccinate lyase

[tca:658327](http://www.kegg.jp/dbget-bin/www_bget?tca:658327) ATP-dependent 6-phosphofructokinase

[tca:658584](http://www.kegg.jp/dbget-bin/www_bget?tca:658584) glutamate synthase 1 [NADH], chloroplastic

[tca:658917](http://www.kegg.jp/dbget-bin/www_bget?tca:658917) delta-1-pyrroline-5-carboxylate synthase

[tca:659253](http://www.kegg.jp/dbget-bin/www_bget?tca:659253) L-threonine ammonia-lyase

[tca:662431](http://www.kegg.jp/dbget-bin/www_bget?tca:662431) branched-chain-amino-acid aminotransferase, cytosolic

[tca:663218](http://www.kegg.jp/dbget-bin/www_bget?tca:663218) D-3-phosphoglycerate dehydrogenase

[tca:663325](http://www.kegg.jp/dbget-bin/www_bget?tca:663325) probable phosphoserine aminotransferase

 **[tca04068 FoxO signaling pathway - Tribolium castaneum (red flour beetle)](http://www.kegg.jp/kegg-bin/show_pathway?148760529482229/tca04068.args) (**[**8**](javascript:display('tca04068'))**)**

[tca:100142381](http://www.kegg.jp/dbget-bin/www_bget?tca:100142381) insulin-like peptide

[tca:659674](http://www.kegg.jp/dbget-bin/www_bget?tca:659674) serine/threonine-protein kinase NLK

[tca:660037](http://www.kegg.jp/dbget-bin/www_bget?tca:660037) DER; epidermal growth factor receptor

[tca:660418](http://www.kegg.jp/dbget-bin/www_bget?tca:660418) G2/mitotic-specific cyclin-B3

[tca:660822](http://www.kegg.jp/dbget-bin/www_bget?tca:660822) polo; polo

[tca:661524](http://www.kegg.jp/dbget-bin/www_bget?tca:661524) InR; insulin-like receptor

[tca:664090](http://www.kegg.jp/dbget-bin/www_bget?tca:664090) forkhead box protein O

[tca:664271](http://www.kegg.jp/dbget-bin/www_bget?tca:664271) ilr2; insulin-like receptor

 **[tca04213 Longevity regulating pathway - multiple species - Tribolium castaneum (red flour beetle)](http://www.kegg.jp/kegg-bin/show_pathway?148760529482229/tca04213.args) (**[**8**](javascript:display('tca04213'))**)**

[tca:100142381](http://www.kegg.jp/dbget-bin/www_bget?tca:100142381) insulin-like peptide

[tca:107397949](http://www.kegg.jp/dbget-bin/www_bget?tca:107397949) adenylate cyclase type 8-like

[tca:655273](http://www.kegg.jp/dbget-bin/www_bget?tca:655273) adenylate cyclase type 8

[tca:661438](http://www.kegg.jp/dbget-bin/www_bget?tca:661438) rut; Ca(2+)/calmodulin-responsive adenylate cyclase

[tca:661524](http://www.kegg.jp/dbget-bin/www_bget?tca:661524) InR; insulin-like receptor

[tca:662326](http://www.kegg.jp/dbget-bin/www_bget?tca:662326) heat shock 70 kDa protein cognate 2

[tca:664090](http://www.kegg.jp/dbget-bin/www_bget?tca:664090) forkhead box protein O

[tca:664271](http://www.kegg.jp/dbget-bin/www_bget?tca:664271) ilr2; insulin-like receptor
