# Supplementary material for: Correction to: Multiple functions of CREB-binding protein during postembryonic development: identification of target genes
Source: BMC Genomics. 2018 Aug 6;19:584. doi: 10.1186/s12864-018-4939-8 (PMC6080369; doi:10.1186/s12864-018-4939-8)
Supplement: Supplementary file 1 — Sequence of primers used in the experiments. Figure S1. Checking the knockdown efficiency in T. castaneum larvae and cDNA library preparation for RNA seq. Figure S2. Normalization of RNA-seq data. Figure S3. Histogram presentation of GO ontology classification with 1306 genes that were downregulated in T. castaneum larvae after CBP knockdown. Figure S4. Epi-factor domains within the downregulated genes (1306) after CBP knockdown in T. castaneum larvae. Figure S5. KEGG pathway analysis. Figure S6. Correlation of gene expression levels of 20 selected genes by comparing both qPCR and RNA-seq data. Supporting Information S1. KEGG pathway analysis output. (PDF 892 kb) [file 12864_2018_4939_MOESM1_ESM.pdf]

Multiple functions of CREB-binding protein during postembryonic development: Identification of target genes

*Amit Roy, Smitha George and Subba Reddy Palli**

Department of Entomology, College of Agriculture, University of Kentucky, Lexington, KY 40546, USA

List of materials included

Table S1: Sequences of primers used in the experiments.

Figure S1: Checking the knockdown efficiency in *T.castaneum* larvae and cDNA library preparation for RNA seq.

Figure S2: Normalization of RNA-seq data.

Figure S3: Histogram presentation of GO ontology classification with 1306 genes that were downregulated in *T.castaneum* larvae after CBP knockdown.

Figure S4: Epi-factor domains within the downregulated genes (1306) after CBP knockdown in *T.castaneum* larvae.

Figure S5: KEGG pathway analysis.

Figure S6: Correlation of gene expression levels of 20 selected genes by comparing both qPCR and RNA-seq data.

Supporting Information S1: KEGG pathway analysis output.

Excel file S1: Details of 1306 genes downregulated by CBP RNAi.

Excel file S2: Details of 52 genes identified after k-mer clustering.

Table S1: Sequences of primers used in the experiments.

Gene Name	Direction	Primer sequence(5’-3’)
qRT-PCR primers		
HSP90	Forward	GCGCTAAGTGAAGAGCTAAGA
	Reverse	ATGCACACACGAACAAATCAC
4EBP	Forward	ATCACCGATGGCAAGACAAGTGAC
	Reverse	ATGGCAGTTCAGAAGGGTCGTTGA
CBP	Forward	GGTCCCGATGGTAAGAAGAAAG
	Reverse	CCGAGAGATCATTACCCGTTTG
G13402	Forward	ACTGTGCCGAGTTTAGGA
	Reverse	GAATGCCAGTGGGTCAGG
Kr-h1	Forward	GGCTGCAGACGACTTTCTTTA
	Reverse	GCCGGAAATGGTCGGTTATTA
E75A	Forward	GAAATCGCGTCCAAGTG
	Reverse	GAAGGAAGTTCAATGGC
E75B	Forward	ATGCAGACCGCCACCATCG
	Reverse	CGGGGATGGAGCTGGAGG
Bromodomain protein 2B	Forward	GACAGGCCGATATAACTCAC
	Reverse	ACTCGCTTCTCCCTATCC
FTZ-F1	Forward	TGCGAGGAATCACAAACAAG
	Reverse	TGTGACGTTTGCTCGAAGAC
Rp49	Forward	TGACCGTTATGGCAAACTCA
	Reverse	TAGCATGTGCTTCGTTTTGG
Apolipophorins	Forward	GGGAGATTGCCGATGTTAG
	Reverse	CTTGGCTTGGACCTTGTAG
Forkhead box protein O	Forward	CACGGTCCTAGACGACTTA
	Reverse	CGAGACTTCCCTCCATACT
Hexamerin 1B	Forward	CTCCCGCGAGATCTACAA
	Reverse	GGGAAACCGAACCAGAAC
Krueppel-like factor 6	Forward	GCCTGACTCCAAACGAAG
	Reverse	GCACTTGTACGGCTTCTC
Nuclear receptor corepressor 1	Forward	GCTGTGAGGAACCAATGAG
	Reverse	CGCCGACGAATCGTAATC
Fatty acid synthase	Forward	CCTCGCTGTTGTCCTAAAG
	Reverse	CTCGAGACTTGGTCCAGATA
Chitinase 10	Forward	GACTCGCAAGGCGATAAG
	Reverse	GGGTACTCCCAGTCCAAA
CYP4g7	Forward	GGTCAAGGAAACGGAAGAG
	Reverse	GCCTCGACCATGAAATCC
CYP4g14	Forward	CTTGGAACGGTGTCTTATGG
	Reverse	CGGCAGGAACAGTGTAATC
Ecdysone -induced protein 74EF	Forward	CGATACATTCTCCCGTAGGA
	Reverse	GAGGTGAAGGGTTGGTAGA
TcJhe	Forward	ACTGGAAACCACGAAACG
	Reverse	TAGCCGAAGTCAGGGAAA
Nuclear receptor coactivator 1(SRC)	Forward	CGTCGCCTACGATCACACT
	Reverse	AGCTCGCCTGGTTGTAGTTG
Methoprene-tolerant	Forward	GGGAAAGCAAAGGATCATCA
	Reverse	AAGGCCTTCTTGCTCACTCA
BR-c	Forward	CACAACACTTCTGTCTGCGGTG
	Reverse	CACAGGGTGTTTGCAAGGAG
ECR	Forward	GATGGATGGCGAAGATCAGT
	Reverse	ACTTCGCTGGAACATGCTTT
HR4	Forward	ATAGAACAGCTCCGGCAAGA
	Reverse	TGCCGCAGATTGTATCTCTG
dsRNA primers		
CBP	Forward	CCTCAGACTCCTCAGCTTACTA
	Reverse	GAGCTGAAGTTGGCGTAAATTG
Chip Assay		
Kr-h1	Forward	GGCTGCAGACGACTTTCTTTA
	Reverse	GCCGGAAATGGTCGGTTATTA
G13402	Forward	GACTGTCATCCACAGGGAAA
	Reverse	CAAGGTGTCCTGCAACATTATC
4EBP	Forward	ACTGCCGATGCAAGTCAA
	Reverse	CAGTACGAACGGAGACCATAAC
HSP90	Forward	GCGCTAAGTGAAGAGCTAAGA
	Reverse	ATGCACACACGAACAAATCAC

Figure S1

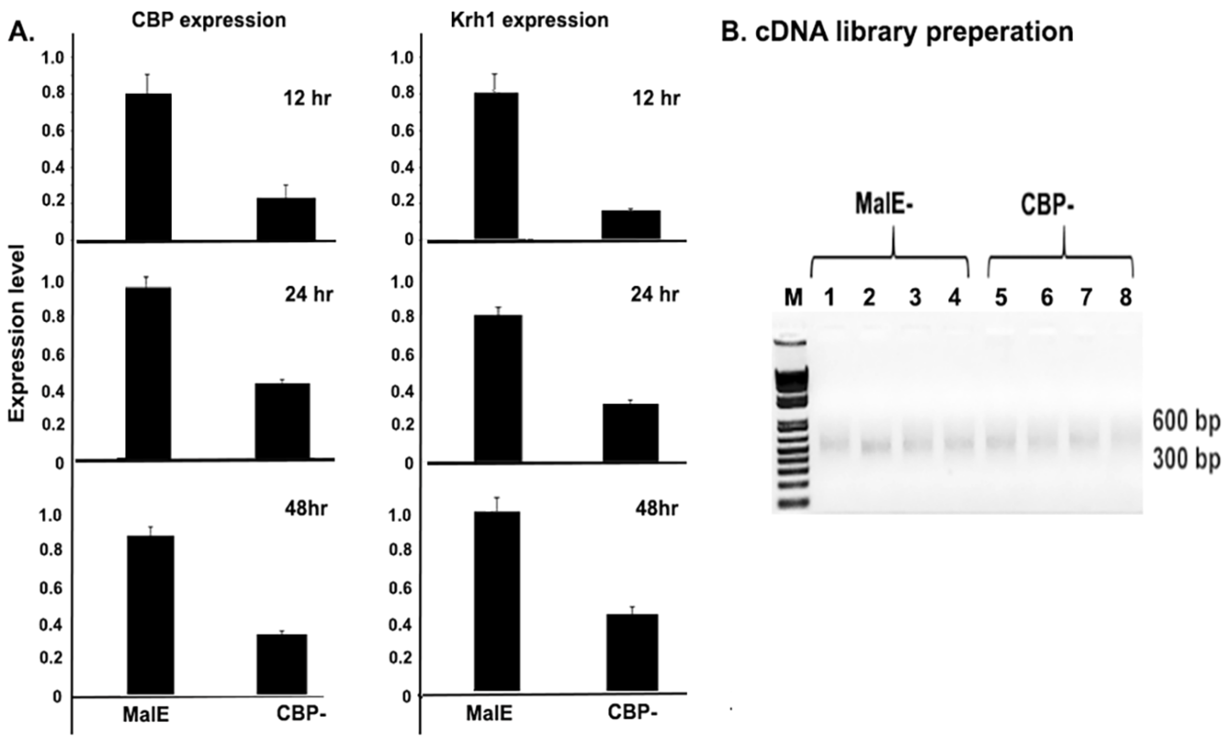

Figure S1: **Checking the knockdown efficiency in *T.castaneum* larvae and cDNA library preparation for RNA seq.** (A) qPCR based expression analysis of CBP and Kr-h1 gene expression after injection of dsmalE and dsCBP at different time points after injection. RNA samples from 12 hr time point are taken for downstream cDNA library preparation and sequencing. (B) The outcome of RNA-seq library preparation. 1.5% agarose gel showing the amplicon size of the prepared cDNA libraries after size selection.

Figure S2

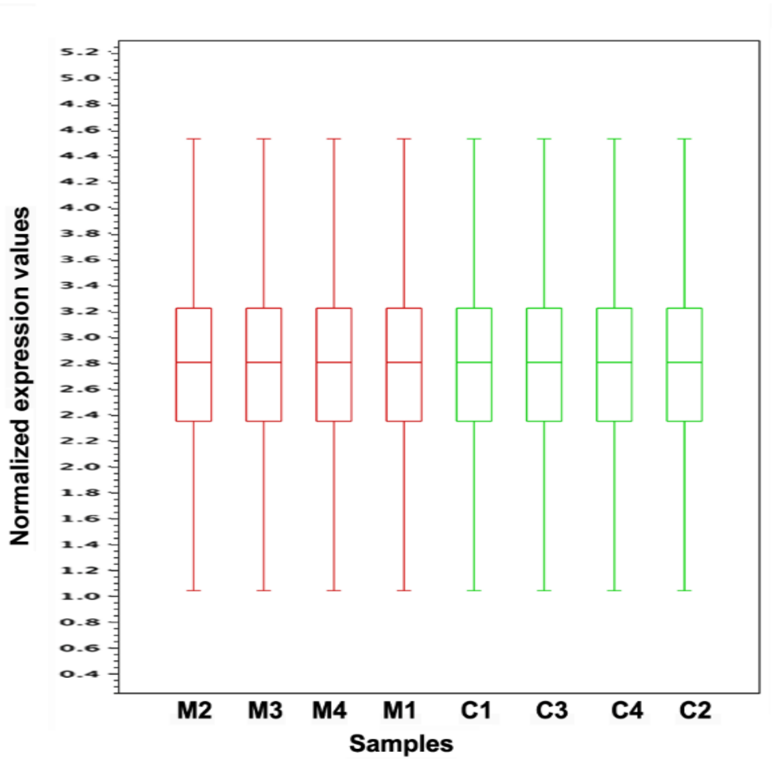

Figure S2: Normalization of RNA-seq data. Box-plot illustrating a high level of expression normalization in control (dsmalE injected) and CBP (dsCBP injected samples.

Figure S3

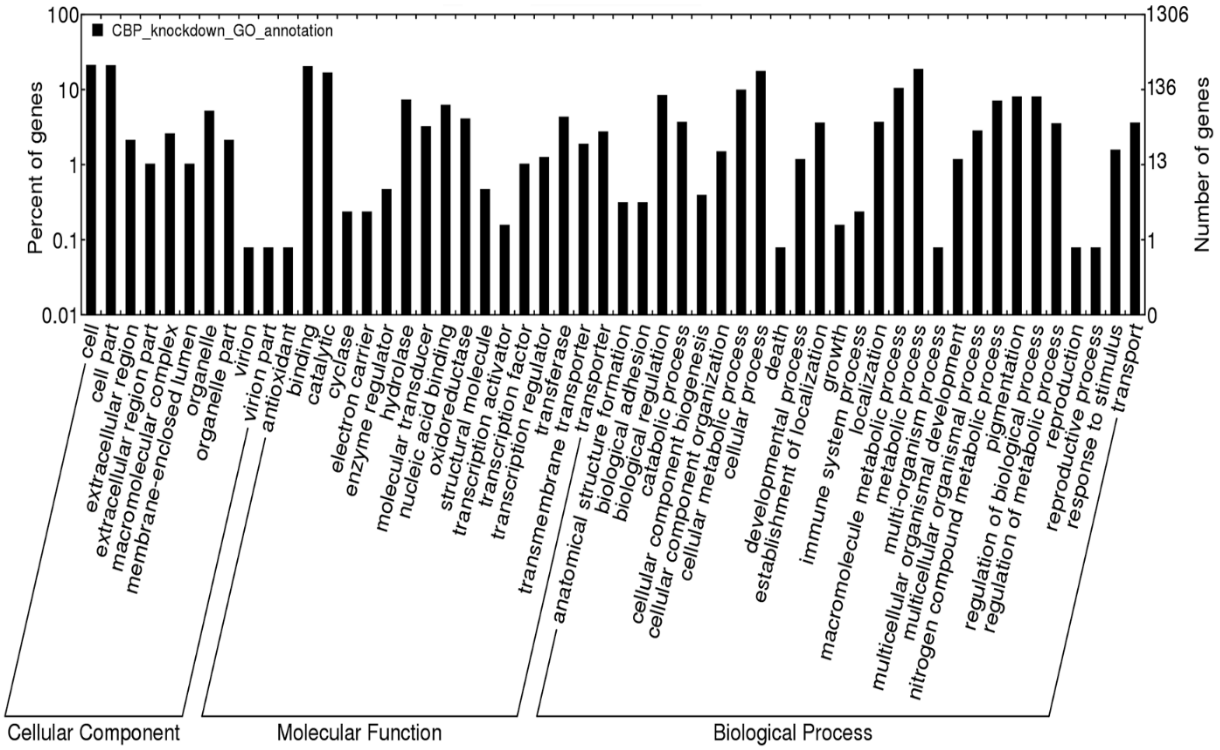

Figure S3: Histogram presentation of GO ontology classification with 1306 genes that were downregulated in *T.castaneum* larvae after CBP knockdown. Classification and functional distribution of the selected 1306 unigenes were represented according to the three major classifications of gene ontology: Biological Process, Molecular Function and Cellular Component.

Figure S4

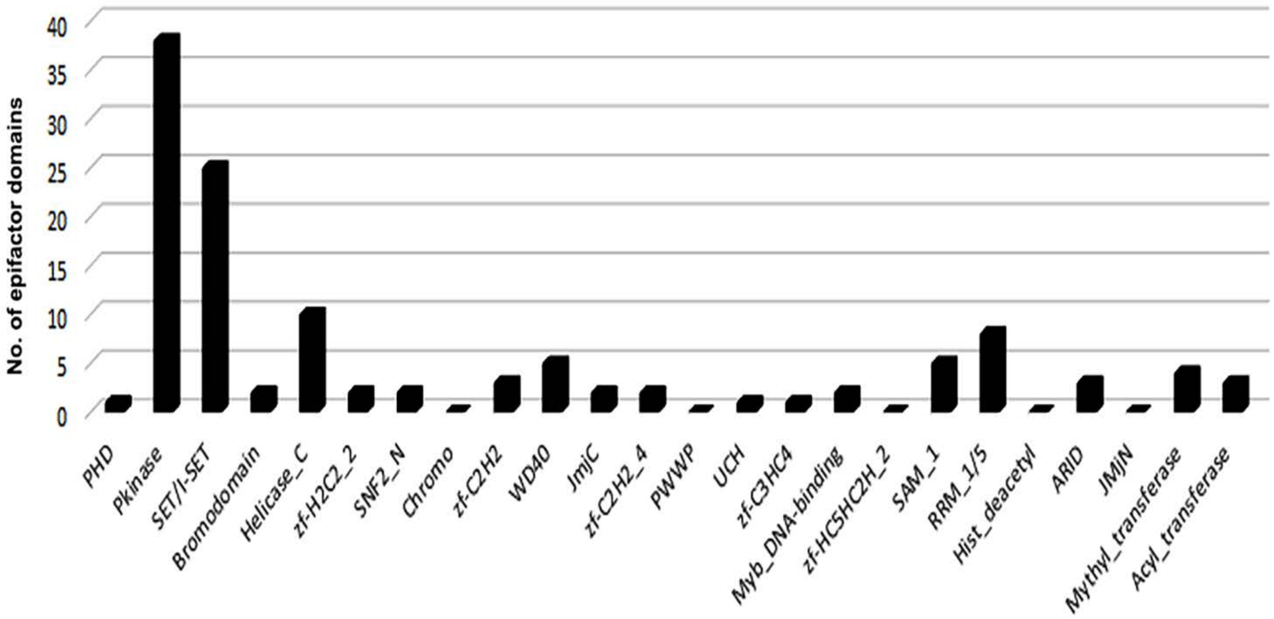

Figure S4: **Epi-factor domains within the downregulated genes (1306) after CBP knockdown in *T.cascaneum* larvae.** High number different epi-factor domains were observed in the target genes indicating their plausible role in epigenetic modulation in *T. castaenum*.

Figure S5

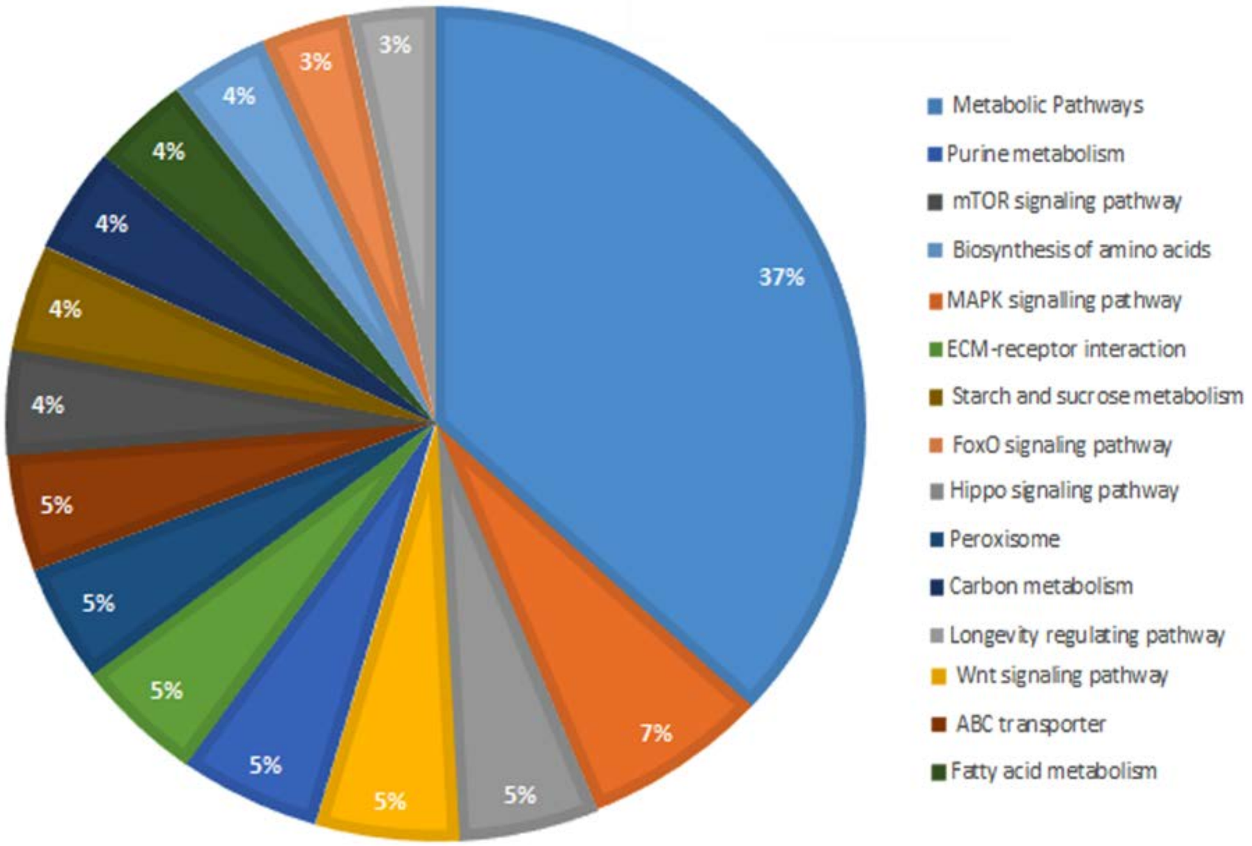

Figure S5: **KEGG pathway analysis.** Listed top 15 physiological processes affected by CBP knockdown in *T.castaneum* larvae.

Figure S6

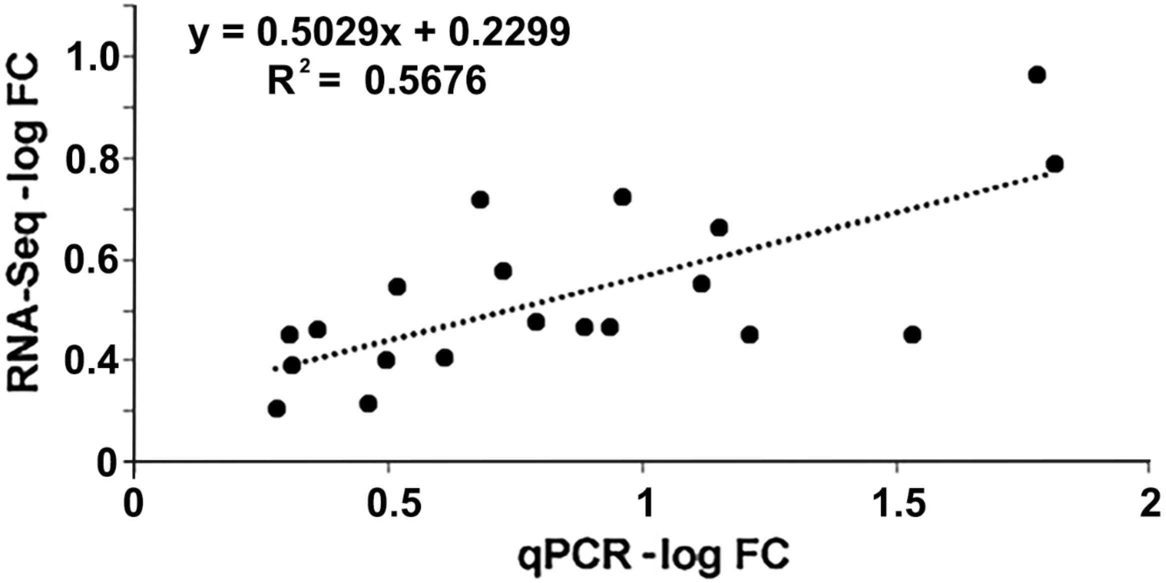

Figure S6: **Correlation of gene expression levels of 20 selected genes by comparing both qPCR and RNA-seq data.** Individual log fold changes obtained by qPCR and RNA-seq for each gene in the sample group.

Supporting Information S1
KEGG pathway analysis output: Top 15 pathways affected by CBP RNAi

List of genes affected by CBP RNAi from each of the pathways given below:

□ tca01100 Metabolic pathways - Tribolium castaneum (red flour beetle) (90)

tca:100141596 glycoprotein-N-acetylgalactosamine 3-beta-galactosyltransferase 1
tca:100142126 hypothetical protein
tca:103312199 sialic acid synthase
tca:103312225 adenosine deaminase CECR1-A
tca:103312380 diacylglycerol kinase eta
tca:641601 Cht5; chitinase 5
tca:652967 Cht10; chitinase 10
tca:654917 alpha-1,3-mannosyl-glycoprotein 4-beta-N-acetylglucosaminyltransferase B
tca:655213 UDP-glucose 4-epimerase
tca:655392 protein henna
tca:655418 sphingomyelin phosphodiesterase
tca:655432 purine nucleoside phosphorylase
tca:655549 nitric oxide synthase, salivary gland
tca:655758 tryptophan 5-hydroxylase 1
tca:656036 glycerol-3-phosphate acyltransferase 1, mitochondrial
tca:656073 chondroitin sulfate synthase 1
tca:656134 argininosuccinate lyase
tca:656241 bifunctional purine biosynthesis protein PURH
tca:656545 amidophosphoribosyltransferase
tca:656564 alanine--glyoxylate aminotransferase 2-like
tca:656621 xanthine dehydrogenase
tca:656820 C-1-tetrahydrofolate synthase, cytoplasmic
tca:656825 peroxiredoxin-6
tca:656849 sarcosine dehydrogenase, mitochondrial
tca:657046 serine--pyruvate aminotransferase, mitochondrial
tca:657084 phospholipase B1, membrane-associated
tca:657239 aminopeptidase N
tca:657312 aminopeptidase N
tca:657315 neutral ceramidase
tca:657406 glycogen phosphorylase
tca:657468 neutral ceramidase-like
tca:657660 DNA polymerase epsilon catalytic subunit A
tca:657686 NADP-dependent malic enzyme
tca:657796 inositol-trisphosphate 3-kinase A
tca:657862 prolyl 4-hydroxylase subunit alpha-2
tca:657963 methylcrotonoyl-CoA carboxylase subunit alpha, mitochondrial
tca:658024 fatty acid synthase
tca:658173 alpha-1,6-mannosyl-glycoprotein 2-beta-N-acetylglucosaminyltransferase
tca:658249 Nag2; beta-N-acetylglucosaminidase NAG2
tca:658327 ATP-dependent 6-phosphofructokinase
tca:658362 acetyl-CoA carboxylase
tca:658558 aldose reductase
tca:658584 glutamate synthase 1 [NADH], chloroplastic
tca:658613 glycine dehydrogenase (decarboxylating), mitochondrial
tca:658917 delta-1-pyrroline-5-carboxylate synthase
tca:658942 NAD(P) transhydrogenase, mitochondrial
tca:658959 putative polypeptide N-acetylgalactosaminyltransferase 9
tca:659029 long-chain-fatty-acid--CoA ligase 5
tca:659179 fatty acid synthase
tca:659253 L-threonine ammonia-lyase
tca:659356 alpha,alpha-trehalose-phosphate synthase [UDP-forming]
tca:659675 DNA primase small subunit
tca:659687 protein O-mannosyltransferase 1
tca:660178 fatty acid synthase
tca:660290 UDP-glucuronosyltransferase 1-7
tca:660388 alpha-aminoadipic semialdehyde synthase, mitochondrial
tca:660529 UDP-N-acetylglucosamine transferase subunit ALG14 homolog

tca:660733 L-xylulose reductase
tca:660813 NADH-ubiquinone oxidoreductase 49 kDa subunit
tca:660841 L-xylulose reductase
tca:660846 UDP-glucuronosyltransferase 2C1
tca:660900 CAD protein
tca:660961 cytosolic non-specific dipeptidase
tca:661002 lactase-phlorizin hydrolase-like
tca:661138 eye-specific diacylglycerol kinase
tca:661312 1-phosphatidylinositol 4,5-bisphosphate phosphodiesterase epsilon-1
tca:661428 Cht8; chitinase 8
tca:661503 propionyl-CoA carboxylase beta chain, mitochondrial
tca:661511 UDP-glucuronosyltransferase 1-9
tca:661583 LanA; laminin subunit alpha
tca:662050 glycogen [starch] synthase
tca:662176 homocysteine S-methyltransferase
tca:662431 branched-chain-amino-acid aminotransferase, cytosolic
tca:662526 glycogen debranching enzyme
tca:662767 N-acetylgalactosaminyltransferase 7
tca:662782 trehalase
tca:662899 fatty acid synthase
tca:663218 D-3-phosphoglycerate dehydrogenase
tca:663325 probable phosphoserine aminotransferase
tca:663399 fructose-1,6-bisphosphatase isozyme 2-like
tca:663547 PCCA; propionyl-CoA carboxylase alpha chain, mitochondrial
tca:663954 alpha amylase
tca:664022 alpha-amylase
tca:664278 gephyrin
tca:664385 alpha-amylase
tca:664389 alpha-amylase-like
tca:664392 alpha-amylase
tca:664486 phosphatidate phosphatase LPIN3
tca:664509 enolase-phosphatase E1
tca:664593 histidine decarboxylase

□ tca04013 MAPK signaling pathway - fly - Tribolium castaneum (red flour beetle) (17)

tca:100141824 Krn; uncharacterized LOC100141824
tca:103312877 Nasrat; uncharacterized LOC103312877
tca:103313241 uncharacterized LOC103313241
tca:655394 ras GTPase-activating protein 1
tca:656565 tyrosine-protein kinase Src64B
tca:659260 thickveins; bone morphogenetic protein receptor type-1B
tca:659567 sev; proto-oncogene tyrosine-protein kinase ROS
tca:660037 DER; epidermal growth factor receptor
tca:660060 lz; lozenge
tca:660328 pros; homeobox protein prospero
tca:660448 myocyte-specific enhancer factor 2
tca:661159 Omb; optomotor-blind-like
tca:661670 hypothetical protein
tca:661922 sprouty; protein sprouty
tca:662162 Peb; pebbled
tca:664119 tumor necrosis factor receptor superfamily member wengen
tca:664510 mitogen-activated protein kinase kinase kinase 11

□ tca00230 Purine metabolism - Tribolium castaneum (red flour beetle) (13)

tca:103312225 adenosine deaminase CECR1-A
tca:107397949 adenylate cyclase type 8-like
tca:655273 adenylate cyclase type 8
tca:655432 purine nucleoside phosphorylase

tca:656241 bifunctional purine biosynthesis protein PURH
tca:656545 amidophosphoribosyltransferase
tca:656621 xanthine dehydrogenase
tca:657274 retained; high affinity cAMP-specific and IBMX-insensitive 3',5'-cyclic phosphodiesterase 8A
tca:657660 DNA polymerase epsilon catalytic subunit A
tca:658906 high affinity cGMP-specific 3',5'-cyclic phosphodiesterase 9A
tca:659510 probable 3',5'-cyclic phosphodiesterase pde-5
tca:659675 DNA primase small subunit
tca:661438 rut; Ca(2+)/calmodulin-responsive adenylate cyclase

□ tca04310 Wnt signaling pathway - Tribolium castaneum (red flour beetle) (13)

tca:103314170 bambi; BMP and activin membrane-bound inhibitor homolog
tca:655266 calcium/calmodulin-dependent protein kinase type II alpha chain
tca:655873 Axn; axis inhibition protein
tca:655955 Fz4; frizzled 4
tca:656250 protein kinase C, brain isozyme-like
tca:656499 Fz2; frizzled 2
tca:656598 Wnt10; protein Wnt-10a
tca:656854 division abnormally delayed protein
tca:657726 Wnt11; protein Wnt-11b-1
tca:659674 serine/threonine-protein kinase NLK
tca:661080 uncharacterized LOC661080
tca:661337 protein prickle-like
tca:661936 Wnt7; protein Wnt-7b

□ tca04391 Hippo signaling pathway - fly - Tribolium castaneum (red flour beetle) (13)

tca:100141824 Krn; uncharacterized LOC100141824
tca:103313241 uncharacterized LOC103313241
tca:656854 division abnormally delayed protein
tca:657914 Myo20; myosin 20
tca:658147 hemicentin-2
tca:658922 Sd; scalloped
tca:659267 protein expanded
tca:659713 FAT; cadherin-related tumor suppressor
tca:660913 LIX1-like protein
tca:661284 partitioning defective 3 homolog B
tca:662006 dachsous; protein dachsous
tca:663662 protein kibra
tca:664119 tumor necrosis factor receptor superfamily member wengen

□ tca04512 ECM-receptor interaction - Tribolium castaneum (red flour beetle) (12)

tca:100141619 collagen alpha-3(IX) chain
tca:100141763 agrin
tca:103313111 collagen alpha-2(IV) chain
tca:657051 laminin subunit gamma-1
tca:658046 laminin subunit alpha-1
tca:659601 integrin alpha-PS1
tca:660112 fibril-forming collagen alpha chain
tca:661297 laminin subunit beta-1
tca:661583 LanA; laminin subunit alpha
tca:662246 synaptic vesicle glycoprotein 2B
tca:663310 collagen alpha-2(IV) chain
tca:663372 dystroglycan

□ tca02010 ABC transporters - Tribolium castaneum (red flour beetle) (11)

tca:100142393 cystic fibrosis transmembrane conductance regulator
tca:100142464 multidrug resistance-associated protein 4-like
tca:107398636 multidrug resistance-associated protein 4-like
tca:656043 ATP-binding cassette sub-family A member 1-like
tca:657736 ATP-binding cassette sub-family A member 2
tca:658871 probable multidrug resistance-associated protein lethal(2)03659
tca:658981 probable multidrug resistance-associated protein lethal(2)03659
tca:660313 ATP-binding cassette sub-family D member 3
tca:660350 ATP-binding cassette sub-family G member 1
tca:660357 probable multidrug resistance-associated protein lethal(2)03659
tca:660659 multidrug resistance-associated protein 4

□ tca04146 Peroxisome - Tribolium castaneum (red flour beetle) (11)

tca:100141869 putative fatty acyl-CoA reductase CG5065
tca:656115 putative fatty acyl-CoA reductase CG5065
tca:656621 xanthine dehydrogenase
tca:656776 putative fatty acyl-CoA reductase CG5065
tca:657046 serine--pyruvate aminotransferase, mitochondrial
tca:659029 long-chain-fatty-acid--CoA ligase 5
tca:659998 putative fatty acyl-CoA reductase CG5065
tca:660187 putative fatty acyl-CoA reductase CG5065
tca:660313 ATP-binding cassette sub-family D member 3
tca:662263 putative fatty acyl-CoA reductase CG8306
tca:663534 putative fatty acyl-CoA reductase CG5065

□ tca00500 Starch and sucrose metabolism - Tribolium castaneum (red flour beetle) (10)

tca:657406 glycogen phosphorylase
tca:659356 alpha,alpha-trehalose-phosphate synthase [UDP-forming]
tca:662050 glycogen [starch] synthase
tca:662526 glycogen debranching enzyme
tca:662782 trehalase
tca:663954 alpha amylase
tca:664022 alpha-amylase
tca:664385 alpha-amylase
tca:664389 alpha-amylase-like
tca:664392 alpha-amylase

□ tca01200 Carbon metabolism - Tribolium castaneum (red flour beetle) (10)

tca:657046 serine--pyruvate aminotransferase, mitochondrial
tca:657686 NADP-dependent malic enzyme
tca:658327 ATP-dependent 6-phosphofructokinase
tca:658613 glycine dehydrogenase (decarboxylating), mitochondrial
tca:659253 L-threonine ammonia-lyase
tca:661503 propionyl-CoA carboxylase beta chain, mitochondrial
tca:663218 D-3-phosphoglycerate dehydrogenase
tca:663325 probable phosphoserine aminotransferase
tca:663399 fructose-1,6-bisphosphatase isozyme 2-like
tca:663547 PCCA; propionyl-CoA carboxylase alpha chain, mitochondrial

□ tca04150 mTOR signaling pathway - Tribolium castaneum (red flour beetle) (10)

tca:100142381 insulin-like peptide
tca:655955 Fz4; frizzled 4
tca:656250 protein kinase C, brain isozyme-like
tca:656499 Fz2; frizzled 2
tca:656598 Wnt10; protein Wnt-10a

tca:657726 Wnt11; protein Wnt-11b-1
tca:660829 sodium-coupled neutral amino acid transporter 9-like
tca:661524 InR; insulin-like receptor
tca:661936 Wnt7; protein Wnt-7b
tca:664271 ilr2; insulin-like receptor

□ tca01212 Fatty acid metabolism - Tribolium castaneum (red flour beetle) (9)

tca:103314796 acyl-CoA Delta(11) desaturase
tca:656186 very-long-chain (3R)-3-hydroxyacyl-CoA dehydratase hpo-8
tca:656478 acyl-CoA Delta(11) desaturase-like
tca:658024 fatty acid synthase
tca:658362 acetyl-CoA carboxylase
tca:659029 long-chain-fatty-acid--CoA ligase 5
tca:659179 fatty acid synthase
tca:660178 fatty acid synthase
tca:662899 fatty acid synthase

□ tca01230 Biosynthesis of amino acids - Tribolium castaneum (red flour beetle) (9)

tca:655392 protein henna
tca:656134 argininosuccinate lyase
tca:658327 ATP-dependent 6-phosphofructokinase
tca:658584 glutamate synthase 1 [NADH], chloroplastic
tca:658917 delta-1-pyrroline-5-carboxylate synthase
tca:659253 L-threonine ammonia-lyase
tca:662431 branched-chain-amino-acid aminotransferase, cytosolic
tca:663218 D-3-phosphoglycerate dehydrogenase
tca:663325 probable phosphoserine aminotransferase

□ tca04068 FoxO signaling pathway - Tribolium castaneum (red flour beetle) (8)

tca:100142381 insulin-like peptide
tca:659674 serine/threonine-protein kinase NLK
tca:660037 DER; epidermal growth factor receptor
tca:660418 G2/mitotic-specific cyclin-B3
tca:660822 polo; polo
tca:661524 InR; insulin-like receptor
tca:664090 forkhead box protein O
tca:664271 ilr2; insulin-like receptor

□ tca04213 Longevity regulating pathway - multiple species - Tribolium castaneum (red flour beetle) (8)

tca:100142381 insulin-like peptide
tca:107397949 adenylate cyclase type 8-like
tca:655273 adenylate cyclase type 8
tca:661438 rut; Ca(2+)/calmodulin-responsive adenylate cyclase
tca:661524 InR; insulin-like receptor
tca:662326 heat shock 70 kDa protein cognate 2
tca:664090 forkhead box protein O
tca:664271 ilr2; insulin-like receptor
